# Stretch regulates alveologenesis and homeostasis via mesenchymal G_αq/11_-mediated TGFβ2 activation

**DOI:** 10.1242/dev.201046

**Published:** 2023-05-12

**Authors:** Amanda T. Goodwin, Alison E. John, Chitra Joseph, Anthony Habgood, Amanda L. Tatler, Katalin Susztak, Matthew Palmer, Stefan Offermanns, Neil C. Henderson, R. Gisli Jenkins

**Affiliations:** ^1^ Centre for Respiratory Research, Translational Medical Sciences, School of Medicine, University of Nottingham, Nottingham, NG7 2RD, UK; ^2^ Respiratory Medicine, Nottingham NIHR Biomedical Research Centre, Nottingham, NG7 2RD, UK; ^3^ Respiratory Medicine, Biodiscovery Institute, University Park, University of Nottingham, Nottingham, NG7 2RD, UK; ^4^ Margaret Turner Warwick Centre for Fibrosing Lung Disease, National Heart and Lung Institute, Imperial College London, London, SW3 6LY, UK; ^5^ Department of Medicine, Division of Nephrology, University of Pennsylvania Perelman School of Medicine, Philadelphia, PA 19104, USA; ^6^ Department of Pathology, Division of Nephrology, University of Pennsylvania Perelman School of Medicine, Philadelphia, PA 19104-4238, USA; ^7^ Department of Pharmacology, Max Planck Institute for Heart and Lung Research, 61231 Bad Nauheim, Germany; ^8^ Centre for Inflammation Research, University of Edinburgh, EH16 4TJ, UK; ^9^ MRC Human Genetics Unit, Institute of Genetics and Molecular Medicine, University of Edinburgh, Edinburgh, EH4 2XU, UK

**Keywords:** Alveologenesis, TGFβ, G_αq/11_, GPCR, Lung development, Cyclical mechanical stretch, Mouse

## Abstract

Alveolar development and repair require tight spatiotemporal regulation of numerous signalling pathways that are influenced by chemical and mechanical stimuli. Mesenchymal cells play key roles in numerous developmental processes. Transforming growth factor-β (TGFβ) is essential for alveologenesis and lung repair, and the G protein α subunits G_αq_ and G_α11_ (G_αq/11_) transmit mechanical and chemical signals to activate TGFβ in epithelial cells. To understand the role of mesenchymal G_αq/11_ in lung development, we generated constitutive (*Pdgfrb-Cre^+/−^;Gnaq^fl/fl^;Gna11^−/−^*) and inducible (*Pdgfrb-Cre/ERT2^+/−^;Gnaq^fl/fl^;Gna11^−/−^*) mesenchymal G_αq/11_ deleted mice. Mice with constitutive G_αq/11_ gene deletion exhibited abnormal alveolar development, with suppressed myofibroblast differentiation, altered mesenchymal cell synthetic function, and reduced lung TGFβ2 deposition, as well as kidney abnormalities. Tamoxifen-induced mesenchymal G_αq/11_ gene deletion in adult mice resulted in emphysema associated with reduced TGFβ2 and elastin deposition. Cyclical mechanical stretch-induced TGFβ activation required G_αq/11_ signalling and serine protease activity, but was independent of integrins, suggesting an isoform-specific role for TGFβ2 in this model. These data highlight a previously undescribed mechanism of cyclical stretch-induced G_αq/11_-dependent TGFβ2 signalling in mesenchymal cells, which is imperative for normal alveologenesis and maintenance of lung homeostasis.

## Teaser

**Summary:** Mesenchymal cell G_αq/11_ signalling regulates myofibroblast function and stretch-mediated TGFβ2 signalling, which are important for alveologenesis and organ homeostasis and could be relevant to both developmental and adult lung disease.

## INTRODUCTION

Normal alveologenesis requires tight spatiotemporal control of numerous molecular signalling pathways, and coordinated crosstalk between multiple cell types. Any perturbation to these complex processes can disrupt alveolar formation, resulting in structural and functional abnormalities in the gas exchange regions of the lungs. Such abnormalities contribute to perinatal death and lifelong lung function disturbances in survivors (Lovering et al., 2014). The alveolar stage is the final phase of lung development, during which primitive pulmonary sacculi are divided by newly formed secondary septa to form mature alveoli. Alveolarisation occurs between 36 weeks gestation and around 6 years of age in humans (Donahoe et al., 2016), and from postnatal day (P) 3 to P30 in mice (Beauchemin et al., 2016; Li et al., 2015; Pozarska et al., 2017); therefore, postnatal exposures and stimuli are key influences in alveolar development. Many pathways that drive normal lung development are also instrumental in adult lung repair (Chanda et al., 2019); therefore, understanding normal lung development could have implications for numerous pulmonary diseases.

Mesenchymal cells include various cell types that are integral to normal developmental processes, and pericytes are perivascular cells widely considered to be mesenchymal precursors in the lung (Barron et al., 2016; Kato et al., 2018; Ricard et al., 2014). Pericytes express platelet-derived growth factor receptor-β (PDGFRβ), PDGFRα, and neural/glial antigen 2 (NG2; also known as CSPG4), among other markers. However, the most specific marker for pericytes is PDGFRβ (Riccetti et al., 2020), and PDGFRβ co-expression with other pericyte markers correlates with the expected location of pericytes in the lung (Henderson et al., 2013). Pericytes migrate and differentiate into parenchymal myofibroblasts in the lung, as well as other mesenchymal cell types. Myofibroblast-driven deposition of extracellular matrix (ECM) proteins, such as collagen and elastin, provide the scaffolds for secondary septation during lung development and lung repair (Mecham, 2018; Mizikova and Morty, 2015). Therefore pericytes, and the mesenchymal cells that are derived from them, are instrumental in alveologenesis and lung homeostasis.

The pleiotropic growth factor transforming growth factor-β (TGFβ) regulates numerous developmental and repair processes, including the proliferation, migration and differentiation of pericytes and other mesenchymal cells (Bartram and Speer, 2004), as well as the generation of ECM. TGFβ signalling is tightly regulated *in vivo* by the production of TGFβ in latent form, and the three mammalian TGFβ isoforms, TGFβ1, TGFβ2 and TGFβ3, must be activated to exert their biological effects. Although it is known that TGFβ signalling is essential for multiple processes in alveolar development and repair (Bartram and Speer, 2004), the mechanisms that control TGFβ activation in alveologenesis are unclear.

Latent TGFβ is activated when a conformational change to the large latent complex alters the relationship between TGFβ and the latency-associated peptide, allowing TGFβ to interact with its receptor. The G protein α subunits G_αq_ and G_α11_ (G_αq/11_) mediate TGFβ activation in response to G protein-coupled receptor (GPCR)–ligand binding as well as mechanical stretch in epithelial cells (John et al., 2016; Xu et al., 2009). GPCR signalling has also been implicated in normal alveologenesis (Funke et al., 2016). Cyclical mechanical stretch (CMS) has been shown to induce TGFβ activation in lung slices via a Rho-associated kinase (ROCK)- and αv integrin-dependent process (Froese et al., 2016), although the contribution to this by individual cell types is unknown. Although stretch secondary to foetal breathing movements *in utero* has been shown to be essential for early lung development (Donahoe et al., 2016), the role of breathing-related CMS specifically in mesenchymal cells in alveolar development and the maintenance of adult alveoli has not been investigated.

We hypothesised that G_αq/11_ would mediate CMS-induced TGFβ activation via ROCK and integrin signalling in mesenchymal cells, and that this would be important in alveologenesis and lung homeostasis. Here, using mesenchymal G_αq/11_ knockout mouse models and an *in vitro* CMS system, we show that mesenchymal G_αq/11_ is essential for normal alveologenesis and maintenance of adult alveoli via CMS-induced TGFβ signalling, but that this occurs in a ROCK- and integrin-independent manner via a pathway likely to involve the TGFβ2 isoform.

## RESULTS

### *Pdgfrb-Cre^+/−^;Gnaq^fl/fl^;Gna11^−/−^* mice are growth restricted and are not viable beyond P24

**Fig. 1.**
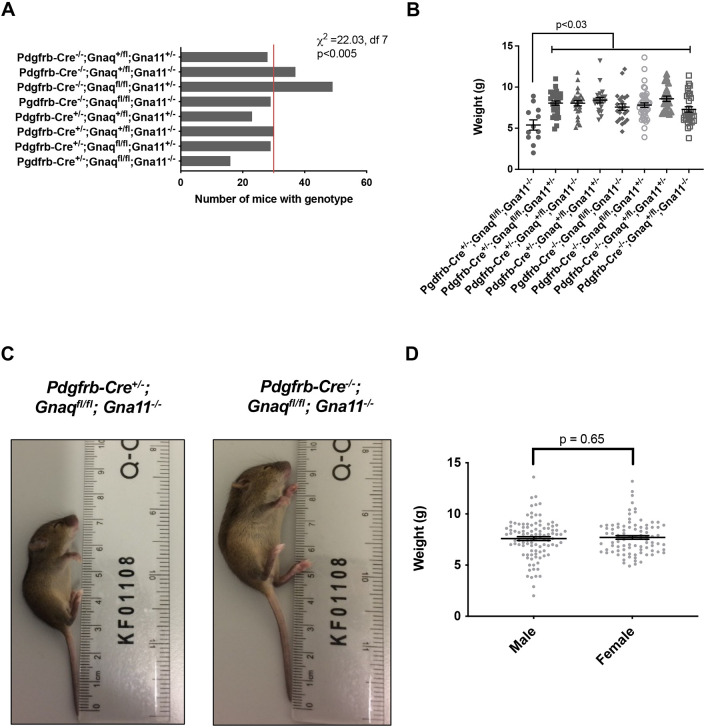
***Pdgfrb-Cre^+/−^;Gnaq^fl/fl^;Gna11^−/−^* mice are growth restricted.** (A) Genotype frequencies from *Pdgfrb-Cre^+/−^*×*Gnaq^fl/fl^;Gna11^−/−^* breeding. Red line indicates the expected frequency for each genotype (*n*=30, 12.5%). Total number of mice born=241, 24 litters, mean litter size 7.4. Chi-squared value (χ^2^)=22.03, degrees of freedom (df)=7, *P*<0.005. (B) Body weights of P14 pups by genotype. Mean±s.e.m.; one-way ANOVA with Tukey's multiple comparisons test; *n*=12 *Pdgfrb-Cre^+/−^*×*Gnaq^fl/fl^;Gna11^−/−^* mice, *n*=21-43 for other genotypes. (C) Photograph of a P14 pup with the *Pdgfrb-Cre^+/−^; Gnaq^fl/fl^; Gna11^−/−^* genotype (left) and a *Pdgfrb-Cre^−/−^; Gnaq^fl/fl^;Gna11^−/−^* control littermate (right). (D) Body weights of all pups from *Pdgfrb-Cre^+/−^*×*Gnaq^fl/fl^;Gna11^−/−^* crosses by sex at P14. Mean±s.e.m.; unpaired two-tailed Student's *t*-test; 88 female and 102 male mice.

To understand whether mesenchymal G_αq/11_ deletion resulted in detrimental effects *in vivo*, gross phenotypes and genotype frequencies of offspring from the *Pdgfrb-Cre^+/−^*×*Gnaq^fl/fl^;Gna11^−/−^* crosses were analysed. Fewer mesenchymal G_αq/11_ knockout (*Pdgfrb-Cre^+/−^;Gnaq^fl/fl^;Gna11^−/−^*) pups reached genotyping age (P14) than was expected (6.6% observed compared with 12.5% expected, Chi squared value=22.03, *P*<0.005; Fig. 1A). Conversely, mice with at least one functional mesenchymal *Gnaq* or *Gna11* allele reached genotyping age at rates closer to the expected Mendelian frequencies (Fig. 1A). Furthermore, *Pdgfrb-Cre^+/−^;Gnaq^fl/fl^;Gna11^−/−^* pups were notably smaller than littermates with at least one intact mesenchymal *Gnaq* or *Gna11* allele. *Pdgfrb-Cre^+/−^;Gnaq^fl/fl^;Gna11^−/−^* animals had a mean weight 1.9-3.2 g lower than all other genotypes (5.4 g versus 7.3-8.4 g, *P*<0.03; Fig. 1B). *Pdgfrb-Cre^+/−^;Gnaq^fl/fl^;Gna11^−/−^* pups were also smaller in physical size compared with control animals (Fig. 1C). There was no sex-related difference in weight across genotypes (Fig. 1D). These findings indicate that mesenchymal G_αq/11_ deletion causes a detrimental developmental phenotype, leading to death *in utero* or in early life.

The first two *Pdgfrb-Cre^+/−^;Gnaq^fl/fl^;Gna11^−/−^* mice from this breeding programme were humanely killed because of poor physical condition at P21 and P24. Therefore, all further analyses were performed in P14 mice, before evidence of ill health was observed. *Gnaq^fl/fl^;Gna11^−/−^* mice develop normally and do not express a phenotype (John et al., 2016); therefore, *Pdgfrb-Cre^−/−^;Gnaq^fl/fl^;Gna11^−/−^* littermates were used as controls for all analyses to ensure that control mice had an identical genotype to the mesenchymal G_αq/11_ knockout mice other than Cre expression, and to facilitate the use of age-matched littermate controls. From here, mice with the *Pdgfrb-Cre^−/−^;Gnaq^fl/fl^;Gna11^−/−^* genotype will be referred to as *Gna11^−/−^* controls.

### *Pdgfrb-Cre^+/−^;Gnaq^fl/fl^;Gna11^−/−^* mice have impaired alveologenesis

**Fig. 2.**
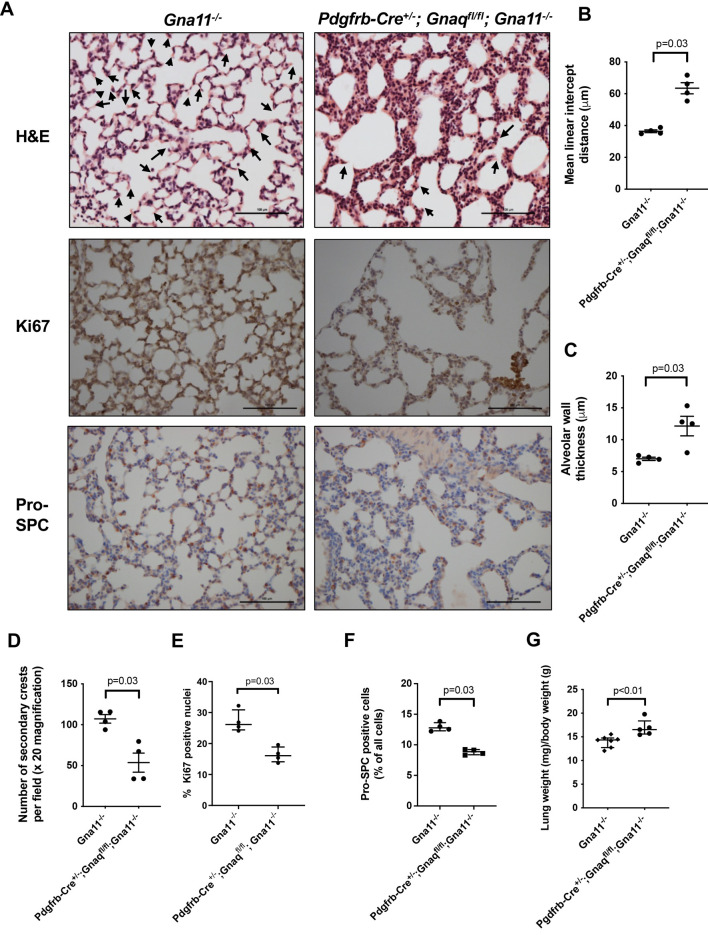
***Pdgfrb-Cre^+/−^;Gnaq^fl/fl^;Gna11^−/−^* mice have abnormal lung appearances characteristic of disturbed alveologenesis.** (A) H&E (top), Ki67 immunohistochemistry (middle) and pro-SPC immunohistochemistry (bottom) staining of lungs from P14 *Gna11^−/−^* (left) and *Pdgfrb-Cre^+/−^;Gnaq^fl/fl^;Gna11^−/−^* mice (right). Arrows on H&E images indicate secondary crests. Images are representative of four mice per group. Scale bars: 100 µm. (B) Mean linear intercept analysis of airspace size in P14 *Gna11^−/−^* and *Pdgfrb-Cre^+/−^;Gnaq^fl/fl^;Gna11^−/−^* mice. Median±interquartile range; *n*=4 mice per group; two-tailed Mann–Whitney test. (C) Alveolar wall thickness in P14 *Gna11^−/−^* and *Pdgfrb-Cre^+/−^;Gnaq^fl/fl^;Gna11^−/−^* mice. Median±interquartile range; *n*=4 mice per group; two-tailed Mann–Whitney test. (D) Quantification of the number of secondary crests per 20× field in P14 *Gna11^−/−^* and *Pdgfrb-Cre^+/−^;Gnaq^fl/fl^;Gna11^−/−^* mice. Median±interquartile range; *n*=4 mice per group; two-tailed Mann–Whitney test. (E) Quantification of Ki67 immunohistochemistry in P14 *Gna11^−/−^* and *Pdgfrb-Cre^+/−^;Gnaq^fl/fl^;Gna11^−/−^* mice, shown as the percentage of Ki67-positive nuclei per 40× magnification field. Median±interquartile range; *n*=4 mice per group; two-tailed Mann–Whitney test. (F) Quantification of pro-SPC immunohistochemistry in P14 *Gna11^−/−^* and *Pdgfrb-Cre^+/−^;Gnaq^fl/fl^;Gna11^−/−^* mice, shown as the percentage of pro-SPC-positive cells per 40× magnification field. Median±interquartile range; *n*=4 mice per group; two-tailed Mann–Whitney test. (G) Relative lung to total body weights in P14 *Gna11^−/−^* and *Pdgfrb-Cre^+/−^;Gnaq^fl/fl^;Gna11^−/−^* mice. Median±interquartile range; *n*=5 *Pdgfrb-Cre^+/−^;Gnaq^fl/fl^;Gna11^−/−^* mice, *n*=6 *Gna11^−/−^* controls; two-tailed Mann–Whitney test.

To understand the role of mesenchymal G_αq/11_ signalling in lung development, the lungs of *Pdgfrb-Cre^+/−^;Gnaq^fl/fl^;Gna11^−/−^* mice and *Gna11^−/−^* controls were examined histologically. *Pdgfrb-Cre^+/−^; Gnaq^fl/fl^;Gna11^−/−^* mouse lungs exhibited clear abnormalities consistent with impaired alveolar development at P14 (Fig. 2A). *Pdgfrb-Cre^+/−^;Gnaq^fl/fl^;Gna11^−/−^* lungs contained enlarged airspaces with a mean linear intercept distance of 63.47 µm compared with 36.43 µm in *Gna11^−/−^* mice (*P*=0.03; Fig. 2B), thickened alveolar walls of 12.2 µm compared with 7.0 µm in *Gna11^−/−^* controls (*P*=0.03; Fig. 2C), and fewer secondary crests (53.7 versus 107.2 per field, *P*=0.03; Fig. 2D) relative to *Gna11^−/−^* littermate controls. Mice with at least one expressed *Gnaq* or *Gna11* allele (i.e. any genotype other than *Pdgfrb-Cre^+/−^;Gnaq^fl/fl^;Gna11^−/−^*) had normal lung histological appearances and similar morphometric measurements that differed from those of *Pdgfrb-Cre^+/−^;Gnaq^fl/fl^;Gna11^−/−^* mice (Fig. S1), indicating that a complete absence of *Gnaq* and *Gna11* in mesenchymal cells is required for the abnormal lung phenotype to be observed. These data also demonstrate that Cre expression or presence of the floxed *Gnaq* alleles alone do not influence the lung phenotype observed in *Pdgfrb-Cre^+/−^;Gnaq^fl/fl^;Gna11^−/−^* mice.

In addition to these structural abnormalities, *Pdgfrb-Cre^+/−^;Gnaq^fl/fl^;Gna11^−/−^* lungs expressed lower levels of the proliferative marker Ki67 (Mki67) than *Gna11^−/−^* controls, with 16% of cell nuclei staining positively for Ki67 in *Pdgfrb-Cre^+/−^;Gnaq^fl/fl^;Gna11^−/−^* lungs compared with 26% in *Gna11^−/−^* controls (*P*=0.03; Fig. 2A,E). Furthermore, *Pdgfrb-Cre^+/−^;Gnaq^fl/fl^;Gna11^−/−^* lungs contained a lower proportion of cells staining positively for the type II epithelial cell marker pro-surfactant protein C (pro-SPC) than *Gna11^−/−^* control lungs, at 8.9% and 12.8% of all cells, respectively (*P*=0.03; Fig. 2A,F).

Finally, *Pdgfrb-Cre^+/−^;Gnaq^fl/fl^;Gna11^−/−^* lungs were heavier relative to total body weight compared with lungs from *Gna11^−/−^* mice (16.5 versus 14.3 mg/g total body weight, *P*<0.01; Fig. 2G), which could be because of elevated lung density or interstitial oedema in these animals. This trend of increased lung weight in *Pdgfrb-Cre^+/−^;Gnaq^fl/fl^;Gna11^−/−^* mice was also observed compared with all other genotypes possible from this breeding strategy (Fig. S1). Overall, these structural, proliferative and cellular differentiation abnormalities indicate a disturbance to alveologenesis in *Pdgfrb-Cre^+/−^;Gnaq^fl/fl^;Gna11^−/−^* mice.

### Myofibroblast differentiation and function is defective in *Pdgfrb-Cre^+/−^;Gnaq^fl/fl^;Gna11^−/−^* mouse lungs

Myofibroblasts are essential for normal alveolar development; therefore, studies were undertaken to assess myofibroblast differentiation and function in *Pdgfrb-Cre^+/−^;Gnaq^fl/fl^;Gna11^−/−^* lungs.

**Fig. 3.**
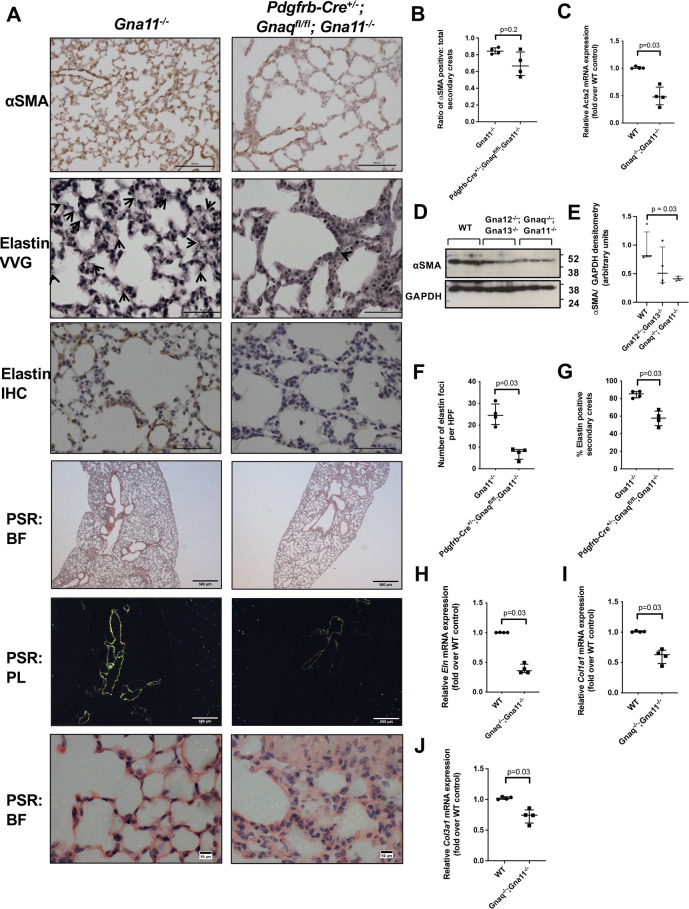
***Pdgfrb-Cre^+/−^;Gnaq^fl/fl^;Gna11^−/−^* mice have reduced lung myofibroblast differentiation and function.** (A) αSMA immunohistochemistry (row 1), elastin Verhoeff–van Gieson (VVG) stain (row 2), elastin immunohistochemistry (row 3) and Picrosirius Red (PSR) staining (rows 4-6) from P14 *Gna11^−/−^* (left) and *Pdgfrb-Cre^+/−^;Gnaq^fl/fl^;Gna11^−/−^* (right) mice. Arrows on elastin images shown elastin foci. PSR images shown are brightfield (BF, rows 4 and 6) and polarised light (PL, row 5). Representative images from four mice per genotype. Scale bars: 100 µm (row 1); 50 µm (rows 2 and 3); 500 µm (rows 4 and 5); 10 µm (row 6). (B) Quantification of the proportion of secondary crests that stained positively for αSMA in P14 *Gna11^−/−^* and *Pdgfrb-Cre^+/−^;Gnaq^fl/fl^;Gna11^−/−^* lungs. Median±interquartile range; *n*=4 mice per group; two-tailed Mann–Whitney test. (C) *Acta2* mRNA expression in WT and *Gnaq^−/−^;Gna11^−/−^* MEFs. Median±interquartile range; *n*=4 per group; two-tailed Mann–Whitney test. (D) Representative western blot showing αSMA expression in WT, *Gna12^−/−^;Gna13^−/−^* and *Gnaq^−/−^;Gna11^−/−^* MEFs. (E) Densitometry of western blots of αSMA expression in WT, *Gna12^−/−^;Gna13^−/−^* and *Gnaq^−/−^;Gna11^−/−^* MEFs. Median±interquartile range; *n*=4; two-tailed Mann–Whitney test. (F) The number of elastin foci per high-powered field (HPF) (40× magnification) in P14 *Gna11^−/−^* and *Pdgfrb-Cre^+/−^;Gnaq^fl/fl^;Gna11^−/−^* lungs. Median±interquartile range; *n*=4 mice per group; two-tailed Mann–Whitney test. (G) The proportion of secondary crests that stained positively for elastin in each high-powered field (40× magnification) in P14 *Gna11^−/−^* and *Pdgfrb-Cre^+/−^;Gnaq^fl/fl^;Gna11^−/−^* lungs. Median±interquartile range; *n*=4 mice per group; two-tailed Mann–Whitney test. (H) *Eln* mRNA expression in WT and *Gnaq^−/−^;Gna11^−/−^* MEFs. Median±interquartile range; *n*=4; two-tailed Mann–Whitney test. (I) *Col1a1* mRNA expression in WT and *Gnaq^−/−^;Gna11^−/−^* MEFs. Median±interquartile range’; *n*=4; two-tailed Mann–Whitney test. (J) *Col3a1* mRNA expression in WT and *Gnaq^−/−^;Gna11^−/−^* MEFs. Median±interquartile range; *n*=4; two-tailed Mann–Whitney test.

Immunohistochemical staining for the myofibroblast marker α-smooth muscle actin (αSMA; Acta2) demonstrated fewer myofibroblasts in the lungs of P14 *Pdgfrb-Cre^+/−^;Gnaq^fl/fl^;Gna11^−/−^* mice compared with *Gna11^−/−^* littermate controls (Fig. 3A). Although overall αSMA staining was decreased in *Pdgfrb-Cre^+/−^;Gnaq^fl/fl^;Gna11^−/−^* lungs, there was no significant reduction in the proportion of αSMA-positive secondary crests compared with *Gna11^−/−^* lungs (0.69 versus 0.84 in controls, *P*=0.2; Fig. 3B).

To investigate whether G_αq/11_ knockout influences myofibroblast differentiation, murine embryonic fibroblasts (MEFs) that were wild-type (WT), G_αq/11_ deficient (*Gnaq^−/−^;Gna11^−/−^*) or G_α12/13_ deficient (*Gna12^−/−^;Gna13^−/−^*) were assessed for αSMA protein and *Acta2* mRNA expression. MEFs with a stable G_αq/11_ knockout had lower *Acta2* mRNA (Fig. 3C) and αSMA protein expression than WT MEFs, whereas MEFs lacking G_α12/13_, another G_α_ subunit family, did not have significantly different αSMA expression compared with WT cells (Fig. 3D,E). This implies a key role for G_αq/11_ signalling in the differentiation of myofibroblasts from mesenchymal precursor cells.

*Pdgfrb-Cre^+/−^;Gnaq^fl/fl^;Gna11^−/−^* lungs also showed evidence of defective myofibroblast synthetic function. *Pdgfrb-Cre^+/−^;Gnaq^fl/fl^;Gna11^−/−^* lungs contained fewer elastin foci (7.4 versus 24.9 foci per field, *P*=0.03; Fig. 3A,F) and fewer elastin-positive secondary crests (57.5% versus 84.8%, *P*=0.03; Fig. 3G) than *Gna11^−/−^* mouse lungs. Furthermore, Picrosirius Red staining revealed that P14 *Pdgfrb-Cre^+/−^;Gnaq^fl/fl^;Gna11^−/−^* mouse lungs contained less collagen than the lungs of *Gna11^−/−^* controls (Fig. 3A). These data were supported by lower *Eln*, *Col1a1* and *Col3a1* mRNA expression in *Gnaq^−/−^;Gna11^−/−^* MEFs than WT MEFs (Fig. 3H-J). These findings imply a failure of myofibroblast differentiation in the lungs of mice lacking mesenchymal G_αq/11_ associated with a reduction in myofibroblast function, leading to a reduction in subepithelial matrix deposition.

### *Pdgfrb-Cre^+/−^;Gnaq^fl/fl^;Gna11^−/−^* mice have abnormal peripheral pulmonary vessels

**Fig. 4.**
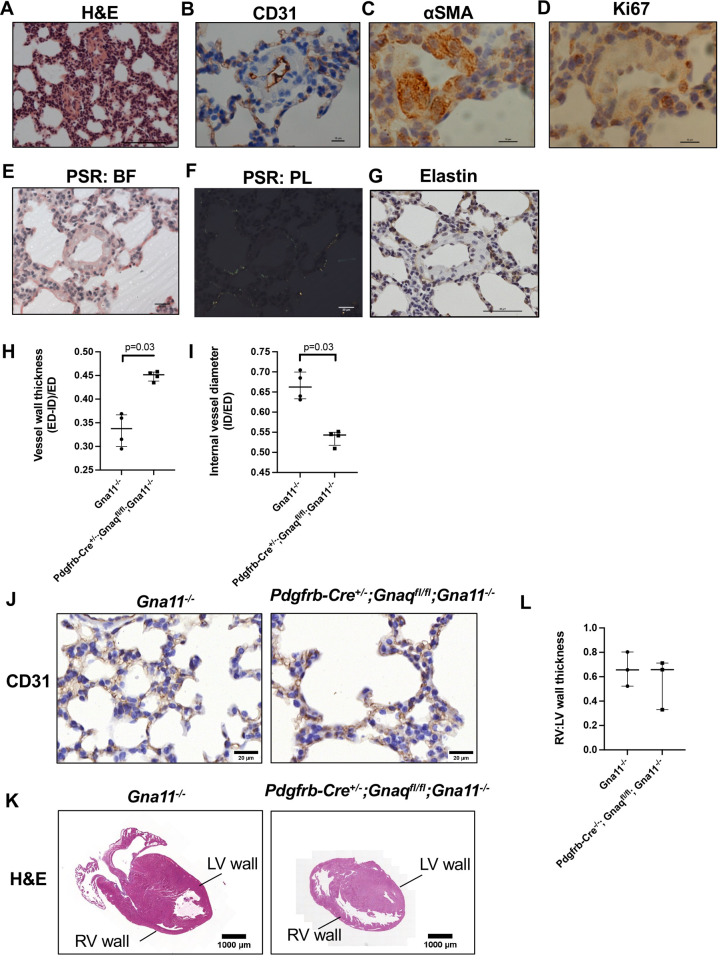
**The lungs of *Pdgfrb-Cre^+/−^;Gnaq^fl/fl^;Gna11^−/−^* mice contain abnormal peripheral pulmonary vessels.** (A-G) Lung sections from P14 *Pdgfrb-Cre^+/−^;Gnaq^fl/fl^;Gna11^−/−^* mice were stained using various techniques: H&E staining (A), CD31 immunohistochemistry (B), αSMA immunohistochemistry (C), Ki67 immunohistochemistry (D), Picrosirius Red staining (PSR) [E,F; the same image is shown using brightfield (BF, E) and polarised light (PL, F) illumination] and elastin immunohistochemistry (G). (H) Quantification of peripheral vessel wall thickness in P14 *Gna11^−/−^* and *Pdgfrb-Cre^+/−^;Gnaq^fl/fl^;Gna11^−/−^* lungs. Vessel wall thickness calculated as (external diameter – internal diameter)/external diameter. Median±interquartile range; *n*=4 mice per group; two-tailed Mann–Whitney test. (I) Quantification of vessel lumen diameter in P14 *Gna11^−/−^* and *Pdgfrb-Cre^+/−^;Gnaq^fl/fl^;Gna11^−/−^* lungs. Vessel lumen diameter calculated as internal diameter/external diameter. Median±interquartile range; *n*=4 mice per group; two-tailed Mann–Whitney test. (J) CD31 immunohistochemistry from P14 *Gna11^−/−^* (left) and *Pdgfrb-Cre^+/−^;Gnaq^fl/fl^;Gna11^−/−^* (right) mice. Representative images from four mice per genotype. (K) H&E staining of representative hearts from P14 *Gna11^−/−^* (left) and *Pdgfrb-Cre^+/−^;Gnaq^fl/fl^;Gna11^−/−^* (right) mice. (L) Right:left cardiac ventricular wall thickness ratios in P14 *Gna11^−/−^* and *Pdgfrb-Cre^+/−^;Gnaq^fl/fl^;Gna11^−/−^* mice. Median±interquartile range; *n*=3 mice per group. LV, left ventricle; RV, right ventricle. Scale bars: 100 µm (A); 10 µm (B-D); 20 µm (E,F,J); 50 µm (G); 1 mm (K).

Pericytes are *Pdgfrb*-expressing cells that originate in the perivascular region (Henderson et al., 2013), and vasculogenesis is an important driver of normal lung development. Therefore, we examined the pulmonary vasculature histologically to assess for abnormalities caused by mesenchymal G_αq/11_ deletion. P14 *Pdgfrb-Cre^+/−^;Gnaq^fl/fl^;Gna11^−/−^* lungs contained markedly abnormal peripheral pulmonary vessels (Fig. 4A-G), with significantly thicker walls (mean ratio of vessel wall thickness:vessel diameter 0.45 versus 0.33 µm, *P*=0.03; Fig. 4H) and reduced vessel lumen diameter (mean ratio of internal vessel diameter:whole vessel diameter of 0.54 compared with 0.67, *P*=0.03; Fig. 4I) than the peripheral pulmonary vessels of *Gna11^−/−^* controls. These vessels consisted of a thin CD31 (Pecam1)-positive endothelial layer (Fig. 4B) surrounded by a thickened αSMA-positive vascular smooth muscle layer (Fig. 4C) without increased proliferation (marked by Ki67; Fig. 4D), indicating that the smooth muscle layer was hypertrophic rather than hyperplastic. These abnormal vessels did not contain significant collagen or elastin layers (Fig. 4E-G). CD31 staining of the alveoli of *Pdgfrb-Cre^+/−^;Gnaq^fl/fl^;Gna11^−/−^* lungs had a similar appearance to those seen in *Gna11^−/−^* lungs at high magnification (Fig. 4J). This argues against there being gross abnormality of the small alveolar vessels; however, this cannot be completely ruled out.

Given that there were some similarities in appearance of the abnormal peripheral pulmonary vasculature in *Pdgfrb-Cre^+/−^;Gnaq^fl/fl^;Gna11^−/−^* lungs to those seen in pulmonary arterial hypertension, we assessed the hearts from these animals for evidence of right ventricular hypertrophy. We found no difference in right:left ventricular wall ratio in *Pdgfrb-Cre^+/−^;Gnaq^fl/fl^;Gna11^−/−^* mice relative to controls (Fig. 4K,L). These data suggest a primary *Pdgfrb^+^* cell-driven defect, rather than secondary pulmonary hypertension due to impaired alveologenesis.

### *Pdgfrb-Cre^+/−^;Gnaq^fl/fl^;Gna11^−/−^* mice have kidney abnormalities

As *Pdgfrb* expression is not exclusive to lung mesenchymal cells, the kidneys, hearts, livers and bowel of *Pdgfrb-Cre^+/−^;Gnaq^fl/fl^;Gna11^−/−^* mice were assessed for extrapulmonary abnormalities.

**Fig. 5.**
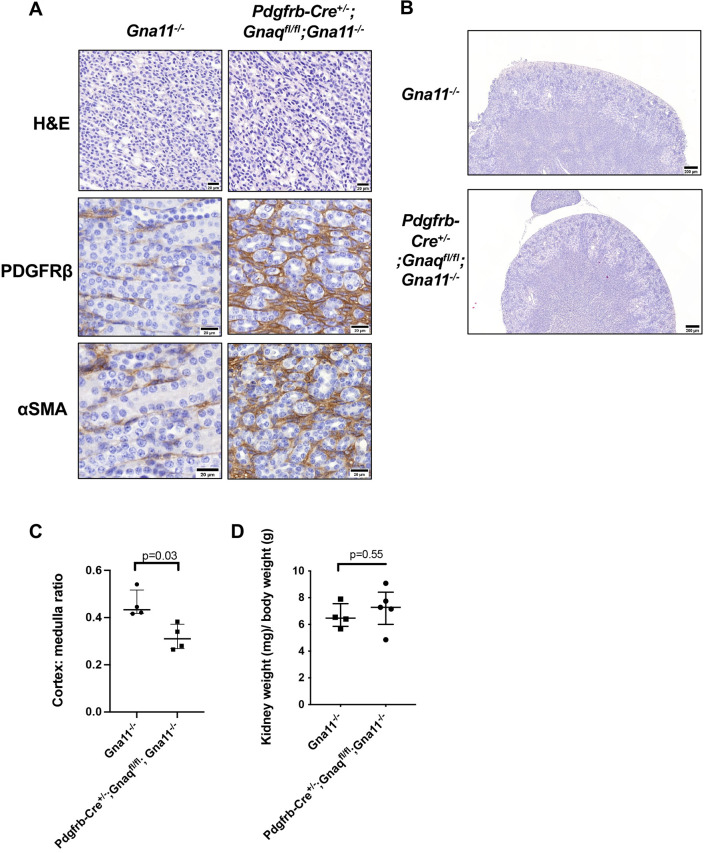
***Pdgfrb-Cre^+/−^;Gnaq^fl/fl^;Gna11^−/−^* mice have kidney abnormalities.** (A) H&E staining, PDGFRβ immunohistochemistry and αSMA immunohistochemistry of renal medulla in P14 *Gna11^−/−^* and *Pdgfrb-Cre^+/−^;Gnaq^fl/fl^;Gna11^−/^* mouse kidneys. Representative images from four mice per genotype. Scale bars: 20 µm. (B) Low-magnification images of H&E staining of P14 *Gna11^−/−^* (top) and *Pdgfrb-Cre^+/−^;Gnaq^fl/fl^;Gna11^−/−^* (bottom) mice. Scale bars: 200 µm. (C) Cortex:medulla ratios of P14 *Gna11^−/−^* and *Pdgfrb-Cre^+/−^;Gnaq^fl/fl^;Gna11^−/^* mice. Median±interquartile range; *n*=4 mice per group; two-tailed Mann–Whitney test. (D) Relative kidney:total body weight in P14 *Gna11^−/−^* and *Pdgfrb-Cre^+/−^;Gnaq^fl/fl^;Gna11^−/−^* mice. Median±interquartile range; *n*=4-5 mice per group; two-tailed Mann–Whitney test.

We observed an expansion and prominence of medullary mesenchymal cells in *Pdgfrb-Cre^+/−^;Gnaq^fl/fl^;Gna11^−/−^* kidneys demonstrated by αSMA and PDGFRβ staining (Fig. 5A), with associated thinning of the cortex (median cortex:medulla ratio 0.31 in *Pdgfrb-Cre^+/−^;Gnaq^fl/fl^;Gna11^−/−^* kidneys and 0.43 in *Gna11^−/−^* controls, *P*<0.03; Fig. 5B,C). The relative kidney to total body weight values of *Pdgfrb-Cre^+/−^;Gnaq^fl/fl^;Gna11^−/−^* mouse kidneys were not different to those of *Gna11^−/−^* controls (median kidney:total body weight ratio 7.3 in *Pdgfrb-Cre^+/−^;Gnaq^fl/fl^;Gna11^−/−^* mice and 6.5 in *Gna11^−/−^* controls, *P*=0.55; Fig. 5D). These data suggest that mesenchymal G_αq/11_ is important in normal kidney development.

*Pdgfrb-Cre^+/−^;Gnaq^fl/fl^;Gna11^−/−^* mice had normal heart, liver and bowel histology (Fig. S2), suggesting that mesenchymal G_αq/11_ signalling is not required for normal heart, liver or bowel development or homeostasis from conception to P14 in mice.

### Mice with mesenchymal G_αq/11_ knockout induced in adulthood have emphysema with altered ECM, but no extrapulmonary abnormalities

**Fig. 6.**
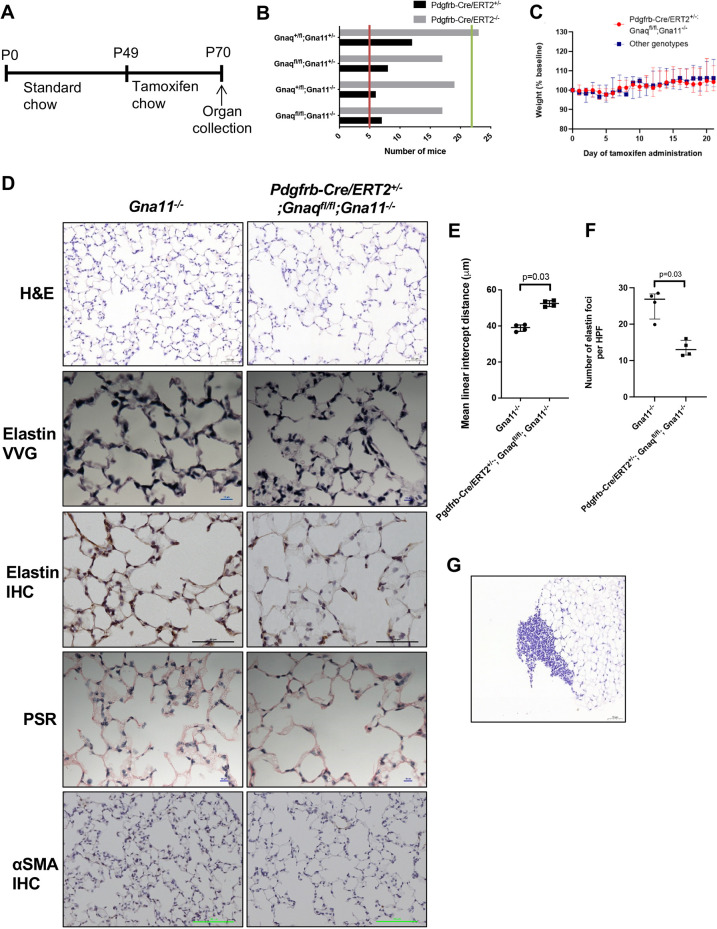
**Mice with mesenchymal G_αq/11_ deletion in adulthood develop emphysema.** (A) Protocol for tamoxifen administration in the *Pdgfrb-Cre/ERT2^+/−^* ×*Gnaq^fl/fl^;Gna11^−/−^* mouse colony. (B) Genotype frequencies from *Pdgfrb-Cre/ERT2^+/−^*×*Gnaq^fl/fl^;Gna11^−/−^* breeding. Red line indicates the expected frequency of *Pdgfrb-Cre/ERT2^+/−^* genotypes (5%; *n*=5), and green line indicates expected frequency *of Pdgfrb-Cre/ERT2^−/−^* genotypes (20%; *n*=22, total *n*=109, 20 litters, mean litter size 5.5). (C) Weights of *Pdgfrb-Cre/ERT2^+/−^;Gnaq^fl/fl^;Gna11^−/^* mice (red) and littermates of all other genotypes (blue) during 21 days of tamoxifen administration. (D) Histology of lungs from *­Gna11^−/−^* control (left) and *Pdgfrb-Cre/ERT2^+/−^;Gnaq^fl/fl^;Gna11^−/−^* (right) mice. IHC, immunohistochemistry; PSR, Picrosirius Red; VVG, Verhoeff–Van Gieson. Representative images from four mice per genotype. Scale bars: 50 µm (H&E); 10 µm (elastin VVG, PSR); 50 µm (elastin IHC); 100 µm (αSMA IHC). (E) Mean linear intercept distance in *Gna11^−/−^* and *Pdgfrb-Cre/ERT2^+/−^;Gnaq^fl/fl^;Gna11^−/−^* mouse lungs. Median±interquartile range; *n*=4 mice per group; two-tailed Mann–Whitney test. (F) Quantification of elastin foci in *Gna11^−/−^* and *Pdgfrb-Cre/ERT2^+/−^;Gnaq^fl/fl^;Gna11^−/−^* mouse lungs. Median±interquartile range; *n*=4 mice per group; two-tailed Mann–Whitney test. (G) Representative image of mononuclear cell infiltrates seen in *Pdgfrb-Cre/ERT2^+/−^;Gnaq^fl/fl^;Gna11^−/−^* mouse lungs.

To assess whether the abnormalities seen in *Pdgfrb-Cre^+/−^;Gnaq^fl/fl^; Gna11^−/−^* mice were related solely to disturbed organ developmental processes or could also affect mature lungs, a tamoxifen-inducible mesenchymal G_αq/11_ knockout model (*Pdgfrb-Cre/ERT2^+/−^;Gnaq^fl/fl^;Gna11^−/−^*) was established in adult mice (Fig. 6A).

Tamoxifen-naïve *Pdgfrb-Cre/ERT2^+/−^;Gnaq^fl/fl^;Gna11^−/−^* mice were born at the expected frequency. According to the supplier, it is expected that 20% of offspring from breeding of the Cre-expressing hemizygous mice with WT mice will express the *Pdgfrb-Cre/ERT2* transgene rather than the 50% Cre-expression rate observed in the germline *Pdgfrb-Cre^+/−^* mouse colony. The frequency of *Pdgfrb-Cre/ERT2^+/−^;Gnaq^fl/fl^;Gna11^−/−^* mice reaching genotyping age was 6.4%, compared with the expected 5% (total number of mice born 109; Fig. 6B). This indicates that having the *Pdgfrb-Cre/ERT2^+/−^;Gnaq^fl/fl^;Gna11^−/−^* genotype, without administration of tamoxifen, does not cause any gross developmental defects.

When a 3-week course of tamoxifen was administered to P49 *Pdgfrb-Cre/ERT2^+/−^;Gnaq^fl/fl^;Gna11^−/−^* mice [*n*=4 (1 female, 3 male)], no detrimental effect to health status was observed compared with littermate controls. Furthermore, *Pdgfrb-Cre/ERT2^+/−^;Gnaq^fl/fl^;Gna11^−/−^* mice gained weight at the same rate as littermate controls with the other genotypes during the tamoxifen protocol (median weight on day 21 of tamoxifen 104.3% of baseline in *Pdgfrb-Cre/ERT2^+/−^;Gnaq^fl/fl^;Gna11^−/−^* mice compared with 106.2% of baseline in other genotypes, *P*=0.71; Fig. 6C). A small reduction in weight was observed early in the tamoxifen protocol that was independent of genotype and was in keeping with a change in diet (Kiermayer et al., 2007). These data suggest that short-term mesenchymal G_αq/11_ knockout does not cause gross physiological disturbances *in vivo*.

Histological analysis revealed that the lungs of *Pdgfrb-Cre/ERT2^+/−^;Gnaq^fl/fl^;Gna11^−/−^* mice treated with tamoxifen had increased airspace size compared with *Gna11^−/−^* controls (mean linear intercept distance 52.5 µm in *Pdgfrb-Cre/ERT2^+/−^;Gnaq^fl/fl^;Gna11^−/−^* mice compared with 39.3 µm in *Gna11^−/−^* controls, *P*=0.03; Fig. 6D,E), suggestive of emphysema. *Pdgfrb-Cre/ERT2^+/−^; Gnaq^fl/fl^;Gna11^−/−^* lungs contained fewer elastin foci than *Gna11^−/−^* controls after 3 weeks of tamoxifen (median number of elastin foci per high-powered field 13.0 in *Pdgfrb-Cre/ERT2^+/−^;Gnaq^fl/fl^;Gna11^−/−^* mice compared with 26.9 in *Gna11^−/−^* controls, *P*=0.03; Fig. 6D,F), similar to the constitutive knockout. In contrast, *Pdgfrb-Cre/ERT2^+/−^;Gnaq^fl/fl^;Gna11^−/−^* lungs did not exhibit altered collagen deposition or evidence of fewer myofibroblasts (αSMA) compared with *Gna11^−/−^* controls (Fig. 6D). Three of the four *Pdgfrb-Cre/ERT2^+/−^;Gnaq^fl/fl^;Gna11^−/−^* mice also exhibited abnormal pulmonary mononuclear cellular aggregates, which were mainly localised at the pleural surfaces (Fig. 6G) and were not observed in littermate control mice. Despite these abnormalities, *Pdgfrb-Cre/ERT2^+/−^;Gnaq^fl/fl^;Gna11^−/−^* mice did not exhibit signs of respiratory distress.

In contrast with *Pdgfrb-Cre^+/−^;Gnaq^fl/fl^;Gna11^−/−^* mice, *Pdgfrb-Cre/ERT2^+/−^;Gnaq^fl/fl^;Gna11^−/−^* mice administered tamoxifen did not exhibit any renal abnormalities on histology (Fig. S3). This implies that mesenchymal G_αq/11_ is needed for normal kidney development, but not maintenance of the normal kidney.

### Cyclical mechanical stretch-induced TGFβ activation in fibroblasts requires G_αq/11,_ but not ROCK or αv or β1 integrins

**Fig. 7.**
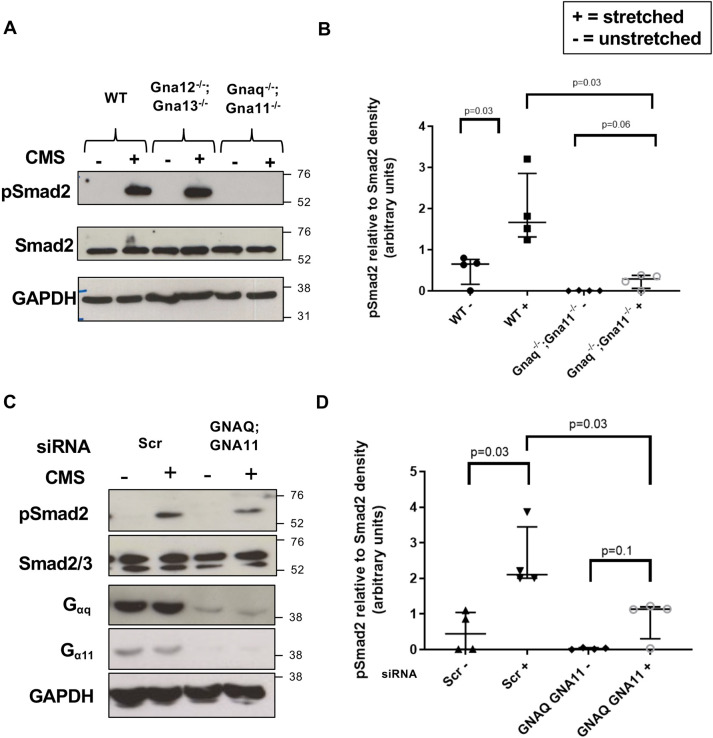
**G_αq/11_ mediates stretch-induced TGFβ signalling in murine and human fibroblasts.** (A) Representative western blot showing pSmad2 expression in WT, *Gna12^−/−^;Gna13^−/−^* and *Gnaq^−/−^Gna11^−/−^* MEFs subject to cyclical mechanical stretch (CMS) (15% elongation, 1 Hz, 48 h). (B) Densitometry of western blots from stretched MEFs shown as pSmad2 relative to Smad2 expression from four independent experiments. Median±interquartile range; *n*=4; two-tailed Mann–Whitney Test. (C) Representative western blot showing pSmad2 expression in HLFs treated with non-targeting (Scr) or *GNAQ* and *GNA11* siRNA then subjected to CMS (15% elongation, 0.3 Hz, 24 h). (D) Densitometry of western blots from stretched HLFs shown as pSmad2 relative to Smad2 expression from four independent experiments. Median±interquartile range; *n*=4; two-tailed Mann–Whitney Test. +, stretched; –, unstretched.

Given the crucial roles of TGFβ in alveolar development, lung repair, and mesenchymal cell migration and differentiation, we investigated the role of mesenchymal G_αq/11_ in a cyclical stretch model of TGFβ activation. Mesenchymal cells with and without intact G_αq/11­_ signalling were subjected to breathing-related CMS and TGFβ signalling was assessed. CMS-induced TGFβ signalling, as assessed by Smad2 phosphorylation, was significantly reduced in *Gnaq^−/−^;Gna11^−/−^* MEFs compared with WT MEFs (Fig. 7A,B). This finding was specific to the G_αq/11_ family of G proteins, as there was no effect of G_α12/13_ knockdown on stretch-induced TGFβ signalling in MEFs (Fig. 7A).

To validate the role of G_αq/11_ in stretch-induced TGFβ signalling in mesenchymal cells across species, human lung fibroblasts (HLFs) with and without siRNA-induced *GNAQ* and *GNA11* knockdown were subjected to breathing-related CMS. *GNAQ* and *GNA11* siRNA led to substantial reductions in both G_αq_ and G_α11_ protein expression in HLFs, and significantly reduced CMS-induced TGFβ signalling compared with scrambled control (Scr) siRNA as measured by phosphorylation of Smad2 (Fig. 7C,D). These data indicate that G_αq/11_ is a key component of CMS-induced TGFβ signalling in both murine and human fibroblasts.

Previous studies have reported that G_αq/11_ induces TGFβ activation via the Rho-ROCK cascade and αv integrins in epithelial cells (Froese et al., 2016; Xu et al., 2009). As αvβ1, αvβ3 and αvβ5 integrins are expressed by myofibroblasts and are involved in TGFβ activation (Pakshir et al., 2020), we utilised chemical inhibition of these integrins and ROCK in our CMS model. When human fibroblasts were subjected to breathing-related CMS in the presence of a ROCK1/2 inhibitor (Y27632), a pan αv integrin inhibitor (CWHM-12) or a β1 integrin-specific inhibitor (NOTT199SS), CMS-induced TGFβ signalling was not reduced (Fig. S4). These data imply the existence of a pathway for CMS-induced TGFβ signalling in mesenchymal cells that requires G_αq/11_, but is independent of ROCK and integrin signalling.

### G_αq/11_ induces TGFβ2 production, which is then available for CMS-induced serine protease-mediated activation

**Fig. 8.**
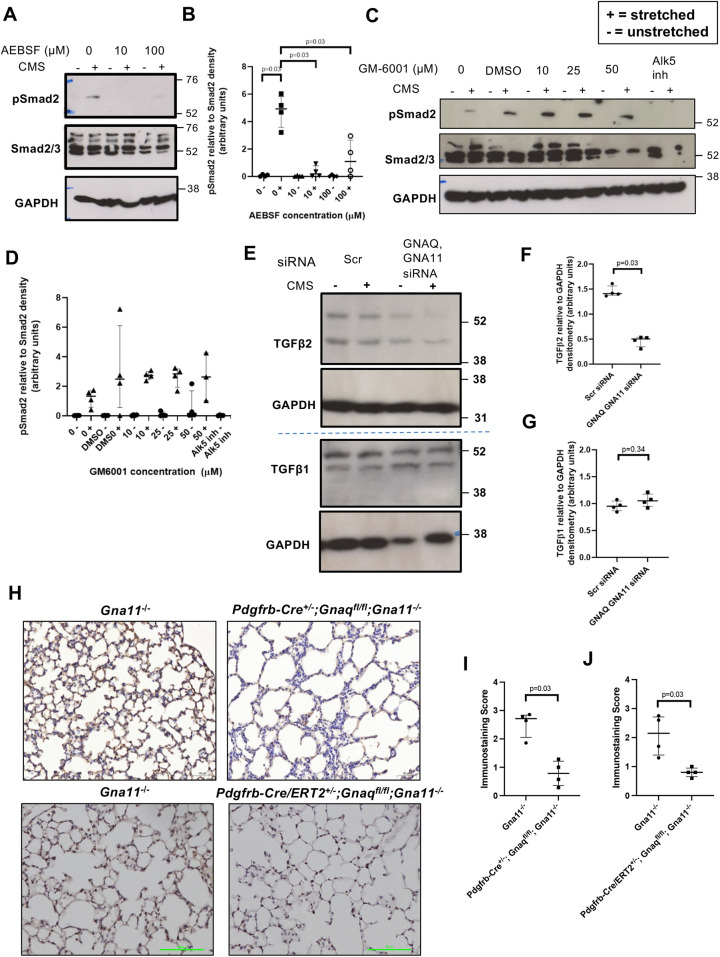
**G_αq/11_ signalling induces the production of TGFβ2, which is then available for stretch-induced serine protease-mediated activation.** (A) Representative pSmad2 western blot of human lung fibroblasts treated with the serine protease inhibitor AEBSF then subjected to CMS (15% elongation, 0.3 Hz, 48 h). (B) Relative pSmad2 to Smad2 densitometry of human lung fibroblasts treated with AEBSF then subjected to CMS. Median±interquartile range; *n*=4; two-tailed Mann–Whitney test. (C) Representative pSmad2 western blot of human lung fibroblasts treated with the MMP inhibitor GM6001 then subjected to CMS (15% elongation, 0.3 Hz, 48 h). (D) Relative pSmad2 to Smad2 densitometry from human lung fibroblasts treated with GM6001 then subjected to CMS. Median±interquartile range; *n*=4; two-tailed Mann–Whitney test. (E) Representative TGFβ2 (top) and TGFβ1 (bottom) western blots of human lung fibroblasts subjected to non-targeting (Scr) or *GNAQ* and *GNA11* siRNA and CMS (15% elongation, 0.3 Hz, 24 h). (F) Relative TGFβ2 to GAPDH densitometry of human lung fibroblasts with and without siRNA-induced *GNAQ* and *GNA11* knockdown. Median±interquartile range; *n*=4; two-tailed Mann–Whitney test. (G) Relative TGFβ1 to GAPDH densitometry of human lung fibroblasts with and without siRNA-induced *GNAQ* and *GNA11* knockdown. Median±interquartile range; *n*=4; two-tailed Mann–Whitney test. (H) TGFβ2 immunohistochemistry on P14 *Pdgfrb-Cre^−/−^;Gnaq^fl/fl^;Gna11^−/−^* control (left) and *Pdgfrb-Cre^+/−^;Gnaq^fl/fl^; Gna11^−/−^* (right) mouse lungs (top row), and tamoxifen-treated P70 *Pdgfrb-Cre/ERT^−/−^;Gnaq^fl/fl^;Gna11^−/−^* control (left) and *Pdgfrb-Cre/ERT2^+/−^;Gnaq^fl/fl^;Gna11^−/−^* mouse lungs. (I) TGFβ2 immunohistochemistry scores of *Pdgfrb-Cre^−/−^;Gnaq^fl/fl^;Gna11^−/−^* control and *Pdgfrb-Cre^+/−^;Gnaq^fl/fl^;Gna11^−/−^* mouse lungs. Median±interquartile range; *n*=4; two-tailed Mann–Whitney test. (J) TGFβ2 immunohistochemistry scores of tamoxifen-treated P70 *Pdgfrb-Cre/ERT2^−/−^;Gnaq^fl/fl^;Gna11^−/−^* control and *Pdgfrb-Cre/ERT2^+/−^;Gnaq^fl/fl^;Gna11^−/−^* mouse lungs. Median±interquartile range; *n*=4; two-tailed Mann–Whitney test. AEBSF, 4-benzenesulfonyl fluoride hydrochloride; Alk5 inh, 50 µM Alk5 inhibitor (SB-525334). +, stretched; –, unstretched.

Proteases can activate latent TGFβ independently of integrins; therefore, we assessed the effect of protease inhibitors in our CMS- induced TGFβ signalling system. A pan serine protease inhibitor 4-(2-aminoethyl) benzenesulfonyl fluoride (AEBSF), decreased CMS-induced Smad2 phosphorylation in HLFs (Fig. 8A,B), whereas the matrix metalloproteinase (MMP) inhibitor GM-6001 had no effect on CMS-induced TGFβ signalling even at high concentrations (Fig. 8C,D). These findings indicate that serine proteases mediate CMS-induced TGFβ signalling in mesenchymal cells.

As TGFβ2 is the only TGFβ isoform that is not activated by integrins (Jenkins, 2008), we hypothesised that breathing-related CMS would predominantly activate the TGFβ2 isoform in mesenchymal cells. Although CMS did not influence TGFβ2 protein expression in HLFs, HLFs with siRNA-induced *GNAQ* and *GNA11* knockdown expressed less TGFβ2 than HLFs with intact G_αq/11_ signalling (Fig. 8E,F), suggesting that G_αq/11_ plays a role in TGFβ2 production. Conversely, TGFβ1 protein expression was not affected by *GNAQ* and *GNA11* knockdown in HLFs (Fig. 8G), suggesting an isoform-specific effect.

To evaluate the role of this CMS-induced TGFβ2 signalling pathway in alveologenesis, we assessed TGFβ2 expression in the lungs of mice from our mouse models. *Pdgfrb-Cre^+/−^;Gnaq^fl/fl^;Gna11^−/−^* lungs had a significantly lower TGFβ2 content than *Gna11^−/−^* control lungs (median immunostaining score 0.8 in *Pdgfrb-Cre^+/−^;Gnaq^fl/fl^;Gna11^−/−^* lungs, compared with 2.7 in *Gna11^−/−^* controls, *P*<0.03; Fig. 8H,I). Similarly, *Pdgfrb-Cre/ERT2^+/−^;Gnaq^fl/fl^;Gna11^−/−^* mouse lungs also had reduced TGFβ2 deposition compared with *Gna11^−/−^* controls after 3 weeks of tamoxifen (median immunostaining score 0.8 in *Pdgfrb-Cre/ERT2^+/−^;Gnaq^fl/fl^;Gna11^−/−^* lungs compared with 2.2 in *Gna11^−/−^* controls, *P*<0.03; Fig. 8H,J). These data demonstrate that lungs lacking mesenchymal G_αq/11_ have less TGFβ2 available for breathing-related CMS-induced activation, and this may be important in alveologenesis and the maintenance of normal lung structure *in vivo*.

### G_αq/11_ influences expression of PDGF signalling components

Platelet-derived growth factor (PDGF) signalling is known to be important in alveolar development, and this pathway interacts with TGFβ signalling in normal development and disease (Gouveia et al., 2017, 2018). We therefore investigated how G_αq/11_ signalling influences the expression of PDGF signalling components in fibroblasts.

**Fig. 9.**
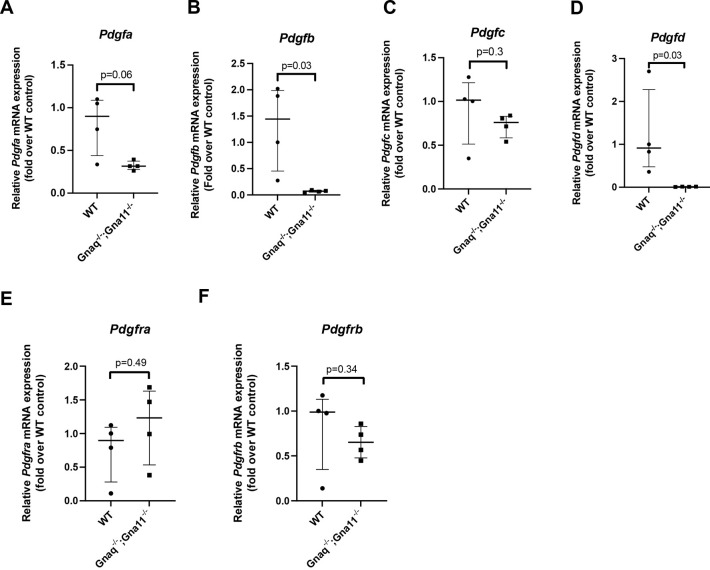
**G_αq/11_ deletion influences expression of some PDGF transcripts in MEFs.** (A-F) Relative mRNA expression of *Pdgfa* (A), *Pdgfb* (B), *Pdgfc* (C), *Pdgfd* (D), *Pgdfra* (E) and *Pdgfrb* (F) in WT and *Gnaq^−/−^;Gna11^+/−^* MEFs. Median±interquartile range; *n*=4; two-tailed Mann–Whitney test.

*Gnaq^−/−^;Gna11^−/−^* MEFs expressed significantly lower levels of *Pdgfb* and *Pdgfd* mRNA compared with WT cells (*P*=0.03; Fig. 9B,D). There was not a statistically significant difference in the expression of *Pdgfa*, *Pdgfc*, *Pdgfra* or *Pdgfrb* mRNA expression between *Gnaq^−/−^;Gna11^−/−^* and WT MEFs (Fig. 9A,C,E,F), although there was a trend to reduced *Pdgfa* expression in *Gnaq^−/−^;Gna11^−/−^* MEFs (*P*=0.06; Fig. 9A). These data imply that mesenchymal G_αq/11_ deletion influences the expression of PDGF signalling components, and thus may regulate PDGF signalling.

## DISCUSSION

In this study, we used mice with a targeted deletion of G_αq/11_ in *Pdgfrb*-expressing cells to demonstrate that mesenchymal G_αq/11_ is essential for the development and maintenance of normal alveoli. Loss of G_αq/11_-mediated signalling in mesenchymal cells caused failure of the myofibroblast differentiation and ECM synthetic function required for alveolar development and the maintenance of the adult lung, and reduced mesenchymal cell TGFβ2 production is a key factor in these processes. In the absence of mesenchymal G_αq/11,_ TGFβ2 is unavailable for activation by CMS-induced serine proteases, thereby diminishing downstream TGFβ signalling in both developing and adult lungs. These findings establish a previously undescribed role for breathing-related CMS in TGFβ2 generation and suggest a role for TGFβ2 in alveolar development and lung homeostasis.

The role of G_αq/11_ in alveolar development has not previously been investigated, primarily because germline G_αq/11_ deletion is embryonically lethal (Offermanns et al., 1998) and murine alveolarisation occurs entirely postnatally (Beauchemin et al., 2016). Cell type-specific *Gnaq* and *Gna11* deletion in neural, cardiovascular and haematological tissues have various manifestations ranging from no phenotype to profound cardiac abnormalities associated with perinatal death (Hoyer et al., 2010; Sassmann et al., 2010; Wettschureck et al., 2007, 2004, 2005, 2001, 2006). However, alveolar abnormalities have not been described in germline or conditional G_αq/11_ knockout mice, suggesting a unique role for mesenchymal G_αq/11_ in alveolar development and maintenance.

We propose that the key mechanisms underlying the abnormal alveologenesis and emphysema in mice with mesenchymal G_αq/11_ deletion present from conception or induced in adulthood, respectively, are failure of myofibroblast differentiation and synthetic function. Both *Pdgfrb-Cre^+/−^;Gnaq^fl/fl^;Gna11^−/−^* and *Pdgfrb-Cre/ERT2^+/−^;Gnaq^fl/fl^;Gna11^−/−^* mice had lower lung elastin deposition than controls. *Pdgfrb-Cre^+/−^;Gnaq^fl/fl^;Gna11^−/−^* lungs also contained fewer myofibroblasts and less collagen compared with controls, and mesenchymal cells lacking G_αq/11­_ express less *Col1a1*, *Col3a1* and *Eln* mRNA than cells with intact G_αq/11_. As myofibroblasts induce secondary septation by depositing ECM proteins, particularly elastin, at the tips of developing secondary septa (Dabovic et al., 2015), and loss of elastin is a key feature of emphysema (Ito et al., 2019; Suki et al., 2012), these data suggest that mesenchymal G_αq/11_-induced myofibroblast differentiation and function are required for alveolar development and homeostasis. In addition, as elastin is a key factor governing lung compliance (Dolhnikoff et al., 1999; Hilgendorff et al., 2012), and mechanical forces themselves may alter availability of elastin-binding sites (Jesudason et al., 2010; Suki et al., 2012), elastin may influence the response to stretch-related forces. Therefore, the impact of mesenchymal G_αq/11_ deletion on elastin deposition and distribution may influence the response to and generation of mechanical forces within the lungs.

Secondary crest myofibroblasts (SCMFs) are known to derive from PDGFRα-expressing precursors (Boström et al., 1996; Li et al., 2018; Lindahl et al., 1997; McGowan et al., 2008); however, the role of PDGRFβ^+^ precursors in the development of SCMFs has not been described. Although this study cannot definitively conclude that PDGFRβ^+^ precursors differentiate into SCMFs, it does show a role for PDGFRβ^+^ cells in alveolarisation. Whether this occurs via direct differentiation of SCMFs from PDGFRβ^+^ precursors, or via paracrine signalling from PDGFRβ^+^ cells, is currently unknown.

*Pdgfrb-Cre^+/−^;Gnaq^fl/fl^;Gna11^−/−^* mouse lungs contained abnormal peripheral pulmonary vessels, with thickened vessel walls and reduced lumen diameter associated with muscularisation of the media (indicated by αSMA staining). These findings could be explained by pulmonary arterial hypertension (PAH), which could relate to hypoxaemia secondary to the profound pulmonary defects, in combination with disturbed GPCR signalling, resulting in vascular remodelling (Cheng et al., 2012; Patel et al., 2018). However, cardiac histology did not show thickening of the right ventricular wall in *Pdgfrb-Cre^+/−^;Gnaq^fl/fl^;Gna11^−/−^* mice, nor was any intimal or adventitial fibrosis observed, findings inconsistent with substantial PAH. A limitation of this study is that assessments such as the Fulton index to assess for right ventricular hypertrophy were not possible and so it is not possible to determine conclusively whether there was any PAH. An alternative explanation for the abnormal vasculature in *Pdgfrb-Cre^+/−^;Gnaq^fl/fl^;Gna11^−/−^* mice could be that altered activity of *Pdgfrb*-expressing cells influences vascular development or the growth, differentiation and activity of constituent cells, such as vascular smooth muscle cells. Furthermore, the observed increased lung weights in *Pdgfrb-Cre^+/−^; Gnaq^fl/fl^;Gna11^−/−^* mice could be linked to interstitial oedema and cardiac malfunction. However, it is not currently possible to define the precise cause of the vascular abnormalities observed.

Altered CMS-induced TGFβ activation is likely to be a key driver of the lung phenotypes observed in *Pdgfrb-Cre^+/−^;Gnaq^fl/fl^;Gna11^−/−^* and *Pdgfrb-Cre/ERT2^+/−^;Gnaq^fl/fl^;Gna11^−/−^* mice. TGFβ drives myofibroblast differentiation, cellular migration and ECM protein production (Harrell et al., 2018), and deficiencies and genetic polymorphisms in TGFβ signalling pathway components have been associated with emphysema (Bonniaud et al., 2004; Celedón et al., 2004; Hersh et al., 2009; Li et al., 2011). Both lung stretch and tightly controlled TGFβ signalling are important for normal lung development and regeneration (Alejandre-Alcázar et al., 2008; Belcastro et al., 2015; Bonniaud et al., 2004; Chen et al., 2005, 2008; Deng et al., 2019; Donahoe et al., 2016; Gauldie et al., 2003; Nakanishi et al., 2007; Pieretti et al., 2014; Sterner-Kock et al., 2002; Vicencio et al., 2004), and CMS has been demonstrated to induce TGFβ signalling in a number of models and organ systems (Froese et al., 2016; Fujita et al., 2010; Furumatsu et al., 2013; John et al., 2016; Maeda et al., 2011; Russo et al., 2018; Wang et al., 2013). Using the same *Gnaq^fl/fl^;Gna11^−/−^* mice used in our study, John et al. described age-related emphysema related to reduced stretch-induced TGFβ signalling in mice lacking G_αq/11_ in type II alveolar epithelial cells (John et al., 2016). Open access RNA-sequencing data on the LungMAP and IPF Cell Atlas databases show that in human and mouse lung, PDGFRβ-positive cells include pericytes, fibroblasts and myofibroblasts (www.ipfcellatlas.com: Kaminski/Rosas dataset; www.lungmap.net: mouse: LungMAP ID LMEX0000001602; human: LungMAP ID LMEX0000004388). We therefore used human lung fibroblasts and murine embryonic fibroblasts to assess the role of mesenchymal G_αq/11_ in CMS-induced TGFβ signalling.

CMS-induced TGFβ signalling in HLFs and MEFs was dependent on serine proteases and independent of αv integrins, contrary to previous work in lung slices and epithelial cells (Froese et al., 2016; Xu et al., 2009). This indicated that TGFβ2, an isoform that is activated by proteases but not integrins (Jenkins, 2008), may be the primary TGFβ isoform activated by mesenchymal cell stretch. G_αq/11_-deficient human lung fibroblasts expressed less TGFβ2, but had unchanged levels of TGFβ1, compared with cells that express G_αq/11_, suggesting a TGFβ isoform-specific effect of G_αq/11_ deletion. These data suggest a pathway in which mesenchymal G_αq/11_ drives TGFβ2 production, which is then available for protease-mediated activation. Although the use of MEFs and HLFs does not precisely recapitulate the fibroblast cell populations present during lung development, these data demonstrate conservation of stretch-induced TGFβ signalling in fibroblasts across species.

This is the first study to propose an isoform-specific role for TGFβ2 in mammalian alveolar development and lung homeostasis. The three TGFβ isoforms are highly expressed during lung development with distinct spatial and temporal expression patterns (Schmid et al., 1991); however, little is known about the specific regulation of TGFβ2 signalling. *Tgfb2^−/−^* mice die shortly after birth from developmental abnormalities distinct from those seen in *Tgfb1^−/−^* or *Tgfb3^−/−^* mice (Kaartinen et al., 1995; Sanford et al., 1997; Shull et al., 1992). *Tgfb2^−/−^* mice have no gross lung morphological abnormalities in late intrauterine gestation; however, collapsed conducting airways are found postnatally (Sanford et al., 1997). Although the *Pdgfrb-Cre^+/−^;Gnaq^fl/fl^;Gna11^−/−^* mice generated in the present study did not share phenotypic features with*Tgfb2^−/−^* mice, it is possible that TGFβ2 production by non-mesenchymal cells is sufficient for normal prenatal development. Additionally, as alveolarisation occurs entirely postnatally in mice, the role of TGFβ2 in alveolar development that we describe could not be observed in *Tgfb2^−/−^* mice owing to perinatal death. Our data demonstrate that loss of mesenchymal G_αq/11_ causes a loss of the precise control of TGFβ signalling in the lungs, resulting in abnormal alveologenesis and loss of lung homeostasis in developed lungs. Further work is required to understand the precise roles of individual TGFβ isoforms in these processes.

The PDGF family is known be important in lung development and regeneration, with PDGF-A being particularly important in alveolar development (Gokey et al., 2021; Gouveia et al., 2018, 2020). We found a trend towards reduced *Pdgfa* expression in MEFs with G_αq/11_ deletion, as well as *Pdgfb* and *Pdgfc*, suggesting that G_αq/11_ signalling may interact with PDGF-related pathways. Postnatal deletion of *Pdgfra*, which encodes the major receptor for PDGF-A, reduces lung *Tgfb2*, but not *Tgfb1*, transcripts (Li et al., 2019), further supporting a role for PDGF signalling in G_αq/11_- and TGFβ2-driven alveolar development and regeneration. However, elastin deposition during alveologenesis may not be dependent on PDGF-A (Gouveia et al., 2020); therefore, PDGF-independent pathways are also likely to be involved in driving the abnormalities in *Pdgfrb-Cre^+/−^;Gnaq^fl/fl^;Gna11^−/−^* and *Pdgfrb-Cre/ERT2^+/−^;Gnaq^fl/fl^;Gna11^−/−^* mouse lungs. As pulmonary mesenchymal cells are predominantly PDGF receptor expressing, rather than PDGF ligand producing (Gouveia et al., 2017), and G_αq/11_ deletion did not alter *Pdgfra* or *Pdgfrb* expression, we hypothesise that mesenchymal G_αq/11_ deletion reduces lung TGFβ2 signalling, which subsequently alters PDGF ligand expression by other cell types. However, it was beyond the scope of this work to dissect the interactions between G_αq/11_, TGFβ2, and PDGF signalling.

As PDGFRβ is a mesenchymal cell marker found outside of the lung, the other organs of *Pdgfrb-Cre^+/−^;Gnaq^fl/fl^;Gna11^−/−^* and *Pdgfrb-Cre/ERT2^+/−^;Gnaq^fl/fl^;Gna11^−/−^* mice were examined histologically. *Pdgfrb-Cre^+/−^;Gnaq^fl/fl^;Gna11^−/−^* kidneys demonstrated expansion and prominence of medullary mesenchymal cells. However, the kidneys of *Pdgfrb-Cre/ERT2^+/−^; Gnaq^fl/fl^;Gna11^−/−^* mice were normal, supporting the hypothesis that abnormalities observed in *Pdgfrb-Cre^+/−^;Gnaq^fl/fl^;Gna11^−/−^* kidneys were developmental in nature.

There are a number of limitations of this study. The poor condition of *Pdgfrb-Cre^+/−^;Gnaq^fl/fl^;Gna11^−/−^* mice limited the analyses to a single time point and precluded the study of CMS *in vivo*. The absence of detailed lineage tracing or single-cell RNA sequencing of the *Pdgfrb-Cre^+/−^;Gnaq^fl/fl^;Gna11^−/−^* mouse lungs means it cannot be confirmed that the abnormalities were driven by any particular mesenchymal cell subtype. *Pdgfrb-Cre^+/−^* mice have been used to investigate the role of pericytes in various organ development and disease models (Diéguez-Hurtado et al., 2019; Eilken et al., 2017; Foo et al., 2006; Gong et al., 2018; He et al., 2020; Henderson et al., 2013; Ivanova et al., 2021; Wang et al., 2017; Zaitoun et al., 2019; Zhang et al., 2021), including a study that used a GFP reporter mouse to demonstrate *Pdgfrb-Cre*-induced gene recombination in lung pericytes during time points relevant to alveolarisation (Kato et al., 2018). However, previous studies have shown that *Pdgfrb-Cre-*induced gene recombination can occur in other cell types, including myofibroblasts induced by injury, fibroblasts, smooth muscle cells and renal interstitial cells (Cuttler et al., 2011; Gong et al., 2018; Henderson et al., 2013; Schiessl et al., 2018; Ulvmar et al., 2016; Wang et al., 2019; Zou et al., 2018). Furthermore, *Pdgfrb* expression may vary at different stages of organ development and cellular differentiation (Salter et al., 2019), and it is possible that altered gene expression in a *Pdgfrb*-expressing precursor cell could influence the characteristics of non-*Pdgfrb*-expressing cells that derive from them. Therefore, although we hypothesise that abnormalities in pericyte activity, differentiation and migration underlie the defective alveologenesis and emphysema in mesenchymal G_αq/11_ knockout mice, the role of other mesenchymal cells in this process cannot be ruled out. However, we can conclude that G_αq/11_ signalling in *Pdgfrb*-expressing cells is important in lung development and homeostasis.

The growth restriction of *Pdgfrb-Cre^+/−^;Gnaq^fl/fl^;Gna11^−/−^* mice may indicate a nutritional deficiency that could have contributed to delayed alveolar development. Although these animals did have renal abnormalities which may have contributed to the poor condition and failure to thrive of *Pdgfrb-Cre^+/−^;Gnaq^fl/fl^;Gna11^−/−^* mice, the bowel appeared normal and mice with mesenchymal G_αq/11_ deletion induced in adulthood had normal kidneys. This suggests a true pulmonary phenotype in mesenchymal G_αq/11_ knockout mice. Additionally, our *in vitro* data provide compelling evidence for a role for mesenchymal G_αq/11_ in a key lung developmental signalling pathway, suggesting that mesenchymal G_αq/11_ deletion generates a true lung developmental phenotype.

Finally, this study has not investigated the role of lung inflammation in mesenchymal G_αq/11_ knockout mice. TGFβ regulates inflammation, and John et al. showed that emphysema in mice with a type II epithelial G_αq/11_ deletion was associated with lung inflammation and M2 macrophage polarisation (John et al., 2016). The mononuclear cellular aggregates in the lungs of mice with mesenchymal G_αq/11_ deletion induced in adulthood could indicate abnormal inflammation in these mice. However, these cellular aggregates were not observed in mice with a germline mesenchymal G_αq/11_ knockout, and it was not possible to fully define the role of inflammation and the immune response in the emphysema observed in *Pdgfrb-Cre/ERT2^+/−^;Gnaq^fl/fl^;Gna11^−/−^* mice in our study.

In conclusion, this is the first study to generate mesenchymal G_αq/11_ deleted mice, and has demonstrated a previously unappreciated signalling pathway for CMS-induced TGFβ2 signalling in murine embryonic and mature human mesenchymal cells that is important for alveologenesis and maintenance of the normal lung. These findings could have implications for the treatment of several conditions associated with dysregulated developmental and repair pathways, including fibrosis and emphysema.

## MATERIALS AND METHODS

### Resources

All reagents and resources are listed in Table S1.

### Husbandry

Mice were housed under specific pathogen-free conditions, with standard food and water available *ad libitum*. All animal experiments were performed in accordance with the Animals (Scientific Procedures) Act 1986, and approved by the Animal Welfare and Ethical Review Board at the University of Nottingham.

### Breeding strategy

For the germline mouse studies, mice with floxed alleles for *Gnaq* and germline deficiency in *Gna11* (*Gnaq^fl/fl^;Gna11^−/−^*) were crossed with mice that express Cre recombinase under the control of the *Pdgfrb* gene (*Pdgfrb-Cre^+/−^*). *Pdgfrb-Cre^+/−^;Gnaq^+/fl^;Gna11^+/−^* offspring from this F1 generation were then bred with *Gnaq^fl/fl^;Gna11^−/−^* founders to produce an F2 generation, including *Pdgfrb-Cre^+/−^;Gnaq^fl/fl^;Gna11^−/−^* mice. The genetic background for all mice was predominantly C57BL6, with a minimum of a six backcross generations. The generation of *Gnaq^fl/fl^;Gna11^−/−^* and *Pdgfrb-Cre^+/−^* mice has been described previously (Foo et al., 2006; Offermanns et al., 1998; Wettschureck et al., 2001).

For the tamoxifen-inducible mouse gene knockout studies, the same breeding strategy was used as for the germline studies but substituting *Pdgfrb-Cre/ERT2^+/−^* mice [029684, *B6.Cg-Tg(Pdgfrb-cre/ERT2)6096Rha/J*, The Jackson Laboratory] for *Pdgfrb-Cre^+/−^* animals. The generation of these mice has been described elsewhere (Gerl et al., 2015).

### Genotyping

Mice were genotyped using DNA isolated from ear-notch biopsies by PCR analysis with allele-specific primers (Eurofins Scientific). Primer sequences were: *Cre* transgene 5′-GCGGTCTGGCAGTAAAAACTATC-3′, 5′-GTGAAACAGCATTGCTGTCACTT-3′ (product 100 bp); internal positive control 5′-CTAGGCCACAGAATTGAAAGATCT-3′, 5′-GTAGGTGGAAATTCTAGCATCATCC-3′ (product 324 bp); *Gna11* WT 5′-AGCATGCTGTAAGACCGTAG-3′, 5′-GCCCCTTGTACAGATGGCAG-3′ (product 820 bp); *Gna11* knockout 5′-CAGGGGTAGGTGATGATTGTG-3′, 5′-GACTAGTGAGACGTGCTACTTCC-3′ (product 450 bp); *Gnaq* WT and floxed alleles 5′-GCATGCGTGTCCTTTATGTGAG 3′, 5′-AGCTTAGTCTGGTGACAGAAG-3′ [products: 600 bp (WT), 700 bp (floxed)]. For *Cre-ERT2*, the following primers were used: 5′-GAACTGTCACCGGGAGGA-3′, 5′-AGGCAAATTTTGGTGTACGG-3′ (400 bp product).

PCR products were analysed by electrophoresis on ethidium bromide-stained agarose gels.

Mice were genotyped at 2 weeks old (P14). Genotype ratios of F2 mice from the *Gnaq^fl/fl^;Gna11^−/−^* and *Pdgfrb-Cre^+/−^* crosses were compared with the expected Mendelian frequency (12.5% per genotype). Similarly, genotype ratios of F2 mice from the *Gnaq^fl/fl^;Gna11^−/−^* and *Pdgfrb-Cre/ERT2^+/−^* crosses were assessed, with an expected frequency of 5% for each Cre-expressing genotype.

### Human cells

For *in vitro* experiments using human lung fibroblasts, cells from four to six donors were used per group. Informed consent was obtained from all donors and work was approved by the East Midlands Nottingham 1 Research Ethics Committee (reference 08/H0407/1). Cells were used at passage 5-6 for all *in vitro* experiments.

HLFs were isolated from donated post-mortem or surgical lung biopsy samples, from male and female donors with and without pulmonary fibrosis. For non-fibrotic fibroblasts, cells were isolated from regions of lung distant from the area of primary diagnosis. Tissue was cut into 1 mm×1 mm pieces and placed 10 mm apart in a 10 cm cell culture dish. Tissue was cultured in DMEM supplemented with 10% foetal calf serum (FCS, Fisher Scientific, 11573397), L-glutamine (4 mM, G7513, Sigma-Aldrich), penicillin (200 units/ml) and streptomycin (0.2 mg/ml) (P4458, Sigma-Aldrich) and amphotericin B (2.5 µg/ml, A2942, Sigma-Aldrich). Fibroblast outgrowth could be seen after 6-8 days. Tissue was removed from the cell culture dish if it became detached, or when cells had reached 80% confluency and were ready for passage. Cells were maintained in a humidified incubator at 37°C, 5% CO_2_/95% air, in Dulbecco's Modified Eagle's Medium (DMEM, Sigma-Aldrich), supplemented with 10% FCS, L-glutamine (4 mM), penicillin (100 units/ml) and streptomycin (0.1 mg/ml).

### Murine cells

WT, *Gna12^−/−^;Gna13^−/−^* and *Gnaq^−/−^;Gna11^−/−^* MEFs were a gift from Professor Stefan Offermanns (Max Planck Institute for Heart and Lung Research, Bad Nauheim, Germany), and their generation has been described elsewhere (Gu et al., 2002; Zywietz et al., 2001). *Gnaq*, *Gna11*, *Gna12* and *Gna13* gene expression was also confirmed in-house prior to these studies (Fig. S5). Cells were maintained in a humidified incubator at 37°C, 5% CO_2_/95% air, in DMEM supplemented with 10% FCS, L-glutamine (4 mM), penicillin (100 units/ml) and streptomycin (0.1 mg/ml).

### *Pdgfrb-Cre^+/−^;Gnaq^fl/fl^;Gna11^−/−^* mouse phenotyping

Litters were observed for signs of ill health daily from birth. Mice were weighed at P14. Male and female mice were included in all analyses. Mice had not undergone any previous procedures. All mice that survived to P14 were phenotyped and had organs collected. Mouse phenotyping analyses were performed by an observer unaware of the genotype. Genotype information was not available to the phenotyping observer until all phenotyping and health status data had been recorded.

For comparisons, *Pdgfrb-Cre^+/−^;Gnaq^fl/fl^;Gna11^−/−^* (mesenchymal G_αq/11_ knockout mice) were compared with littermate *Pdgfrb-Cre^−/−^;Gnaq^fl/fl^;Gna11^−/−^* control mice (referred to as *Gna11^−/−^* controls).

### Tamoxifen-inducible knockouts

*Pdgfrb-Cre/ERT2^+/−^;Gnaq^fl/fl^;Gna11^−/−^* offspring and their littermates were kept under standard conditions until 7 weeks of age (P49), when tamoxifen-containing chow (400 mg/kg tamoxifen citrate, Envigo) was introduced *ad libitum*. Health scoring and weights were measured daily for 3 weeks as tamoxifen was administered. Animals were humanely killed after 3 weeks of tamoxifen administration (at 10 weeks old, P70).

For comparisons, *Pdgfrb-Cre/ERT2^+/−^;Gnaq^fl/fl^;Gna11^−/−^* (tamoxifen-inducible mesenchymal G_αq/11_ knockout mice) were compared with littermate *Pdgfrb-Cre/ERT2^−/−^;Gnaq^fl/fl^;Gna11^−/−^*control mice (referred to as *Gna11^−/−^* controls).

### Organ collection

Mice were humanely killed by intraperitoneal injection of pentobarbital, and organs collected for histological analyses. The lungs were perfused by injecting 40 units/ml heparin sodium in PBS (Wockhardt) into the right ventricle, and inflated by cannulating the trachea and filling the lungs with 10% formalin (VWR) under gravity. The trachea was ligated, and the heart and lungs removed *en bloc*. Livers and kidneys were also collected. Organs were kept in 10% formalin for 24 h before paraffin embedding and sectioning.

### Tissue histology staining

Formalin-fixed, paraffin-embedded tissue sections of lung (3 µm), kidney (3 µm), heart (5 µm) and liver (5 µm) were deparaffinised in xylene and rehydrated in graded alcohols. Haematoxylin and Eosin (H&E), Verhoeff–Van Gieson (elastin) and Picrosirius Red staining were performed as per standard protocols using buffers and stains prepared in-house and mounted in DPX.

#### Staining solutions made in house

The following histology solutions were generated in house: Weigert's iodine [2 g potassium iodide (03124, Sigma-Aldrich), 1 g iodine (326143, Sigma-Aldrich), 100 ml distilled water]; Verheoff's solution [20 ml 5% alcoholic Haematoxylin (H3136, Sigma-Aldrich), 8 ml 10% ferric chloride (157740, Sigma-Aldrich), 8 ml Weigert's iodine]; Van Gieson's solution [5 ml aqueous acid fuschin (F8129, Sigma-Aldrich), 100 ml saturated aqueous picric acid (84512.260, VWR)]; Picrosirius Red solution [0.5 g Direct Red 80 (365548, Sigma-Aldrich), 500 ml saturated aqueous picric acid (84512.260, VWR)]; Weigert's Haematoxylin (1:1 ratio of Weigert's solution A and Weigert's solution B); Weigert's solution A [1% Haematoxylin (H3136, Sigma-Aldrich) in 100% ethanol]; Weigert's solution B [4 ml 30% ferric chloride (157740, Sigma-Aldrich), 1 ml 12 N hydrochloric acid, 95 ml water]; acidified water (5 ml glacial acetic acid, 1 l distilled water); acid/alcohol solution (70% ethanol, 0.1% hydrochloric acid).

#### H&E stain

After being deparaffinised and rehydrated, tissue sections were submerged in Mayer's Haematoxylin (51275, Sigma-Aldrich) for 2 min, acid/alcohol solution for 1 min, then 1% Eosin solution (101411-524, VWR) for 3 min. Sections were rinsed with tap water between each step, then dehydrated and mounted.

#### Elastin (Verhoeff–Van Gieson) stain

Lung sections were deparaffinised and hydrated to distilled water, then stained in Verhoeff's solution for 1 h until the tissue was completely black. Sections were differentiated in 2% ferric chloride (157740, Sigma-Aldrich) until elastin foci were seen on a grey background, incubated in 5% sodium thiosulphate (72049, Scientific Laboratory Supplies) for 1 min, and then washed in running tap water for 5 min. Sections were then counterstained in Van Gieson's solution for 5 min, dehydrated and mounted as above.

#### Picrosirius Red stain

Lung, kidney and heart sections were deparaffinised and hydrated. Nuclei were stained with Weigert's Haematoxylin for 8 min, and then washed in running tap water for 5 min. Sections were incubated in Picrosirius Red for 1 h, washed in two changed of acidified water, then dehydrated and mounted.

### Immunostaining

Tissue sections were deparaffinised in xylene and rehydrated in graded alcohols. Heat-mediated antigen retrieval was performed by boiling sections in a microwave for 20 min in 10 mM citric acid buffer (pH 6.0). Endogenous peroxidase activity was blocked by incubating sections in 3% hydrogen peroxide in methanol for 30 min. Non-specific binding was blocked with 5% goat serum (G9023, Sigma-Aldrich) in 0.1% bovine serum albumin in PBS. Sections were incubated with primary antibody in 5% goat serum overnight at 4°C in a humidified chamber, followed by incubations for 60 min with secondary antibody and 30 min with avidin-biotin complex (SP2001, Vector Laboratories). Sections were then stained with diaminobenzidine (SIGMAFAST, D4418, Sigma-Aldrich), counterstained with Mayer's Haematoxylin (51275, Sigma-Aldrich), and mounted in DPX (06522, Sigma-Aldrich). Slides were washed in PBS between incubation steps.

The following antibodies were used for immunohistochemistry: rabbit anti-αSMA (Abcam, ab5694; 1:500), rabbit anti-CD31 (Abcam, ab182981; 1:2000), rabbit anti-Ki67 (Abcam, ab15580; 1 µg/ml), rabbit anti-pro-surfactant protein C (Sigma-Aldrich, Ab3786; 1:2000), rabbit anti-TGFβ2 (Proteintech, 19999-1-AP; 1:3000), rabbit anti-elastin (Atlas Antibodies, HPA056941; 1:100) and biotinylated goat anti-rabbit IgG (Vector Laboratories, BA1000; 1:200).

### Image quantification

#### Image acquisition

Images of H&E, elastin and immunohistochemical staining were taken using a Nikon 90i microscope and NIS-Elements software v3.2 (Nikon). Polarised light imaging of Picrosirius Red-stained samples was performed using a Zeiss Axioplan microscope (Zeiss) and MicroManager 1.4 software (Vale Lab, UCSF).

#### Staining quantification

For all analyses of histology images, *Pdgfrb-Cre^+/−^ ;Gnaq^fl/fl^;Gna11^−/−^* or *Pdgfrb-Cre/ERT2^+/−^ ;Gnaq^fl/fl^;Gna11^−/−^* mice were compared with *Pdgfrb-Cre^−/−^;Gnaq^fl/fl^;Gna11^−/−^* or *Pdgfrb-Cre/ERT2^−/−^ ;Gnaq^fl/fl^;Gna11^−/−^* littermate controls (referred to as *Gna11^−/−^* controls), respectively. For histological analyses, four animals per genotype were assessed to allow differences in histological appearances to be detected. All image quantification was performed by an observer unaware of the genotype. This observer was not informed of the genotypes until all image quantification data had been recorded.

For quantitative analyses of the lungs, five to ten images were assessed per set of lungs, covering all lobes and avoiding major airways and central blood vessels. All morphometric analyses were performed using NIS Elements software v3.2 (Nikon), with the exception of peripheral pulmonary vessel thickness measurements and kidney measurements, which were performed using CaseViewer 2.3 software (3D Histech).

For quantification of immunohistochemistry and elastin staining, the ‘count’ feature of ImageJ (National Institutes of Health) was used. Elastin foci were identified as black fibres on Verhoeff–Van Gieson staining, and secondary crests were considered elastin positive if they had black staining that was not clearly a cell nucleus on Verhoeff–Van Gieson staining. For immunohistochemistry staining, a cell was counted if it stained brown. Only nuclear DAB staining was counted for Ki67 quantification. For αSMA quantification, the number of αSMA-positive secondary crests per 40× field was counted. For Ki67 and pro-SPC staining, the total number of cells per 40× field was quantified by counting nuclei, and the proportion of Ki67- or pro-SPC-positive cells calculated by dividing the number of stained cells per image by the total number of cells per image.

For quantification of TGFβ2 staining, the following scoring system was used and seven fields (20× magnification) per mouse were analysed: Score 0, no cells stained; Score 0.5, 1-25 cells stained at low intensity; Score 1.0, 1-25 cells stained at high intensity; Score 1.5, 26-50 cells stained at low intensity; Score 2.0, 26-50 cells stained at high intensity; Score 2.5, >50 cells stained at low intensity; Score 3.0: >50 cells stained at high intensity.

#### Morphometry

Mean linear intercept analysis of airspace size was performed as previously described (John et al., 2016). Briefly, 10× magnification images were overlaid with a grid comprising 100 µm squares, and ‘intercepts’ between gridlines and airspace walls counted. The mean linear intercept was calculated by dividing the length of each gridline by the intercept count. For alveolar wall thickness measurements, 40× magnification images were overlaid with five equally spaced horizontal lines and the alveolar wall thickness measured at points where lung tissue crossed each line using the ‘measure’ function of NIS Elements. Mean linear intercept and alveolar wall thickness values were calculated for each mouse from all measurements across all images and data presented as median±interquartile range. For secondary crest counts, 10× magnification images were used and secondary crests counted for each image.

For peripheral vessel wall thickness, ten random peripheral pulmonary vessels per mouse were identified as circular structures lined with a CD31-positive endothelial layer. The ‘measure’ function of CaseViewer was used to measure the external and internal vessel diameters. The external vessel diameter (ED) was defined as the distance from the outermost aspect of the external wall of a vessel to the outermost aspect of the opposite wall, traversing the centre of the vessel lumen. The internal vessel diameter (ID) was defined as the distance from the innermost aspect of the vessel wall to the innermost aspect of the opposite vessel wall, traversing the centre of the vessel lumen. The following equations were used to calculate the vessel wall thickness (VWT) and internal vessel lumen diameter relative to total vessel diameter:

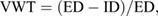


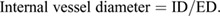
For assessment of right ventricular hypertrophy, the left and right cardiac ventricular wall thickness was measured using CaseViewer, and the right:left ventricular wall thickness ratio calculated.

### Breathing-related cyclical stretch experiments

Cells were seeded at 2×10^5^ cells per well on collagen I-coated Bioflex^®^ 6-well culture plates (3001-C, Dunn Labortechnik) in DMEM supplemented with 10% FCS, L-glutamine (4 mM), penicillin (100 units/ml) and streptomycin (0.1 mg/ml) and allowed to adhere for 24 h. The culture medium was changed to 1% FCS in DMEM with 4 mM L-glutamine for 24 h before stretching commenced. The Flexcell^®^ FX-5000T system (Flexcell International Corporation) was used to apply cyclical stretch to cells *in vitro*, according to the manufacturer's instructions. MEFs were stretched at a frequency of 1 Hz, and HLFs at 0.3 Hz to mimic breathing in the relevant organism. 15% elongation and a sine waveform were used for all cyclical stretch experiments. Cyclical stretch was applied for 48 h, except for experiments using siRNA-induced *GNAQ* and *GNA11* knockdown, for which 24 h of cyclical stretch was used. Unstretched control cells were cultured in identical conditions alongside the Flexcell^®^ apparatus. Cells were lysed in protein lysis buffer (9803, Cell Signaling Technology) supplemented with phosphatase (Phos-Stop, 04906837001, Sigma-Aldrich) and protease (cOmplete Mini, 04693124001, Sigma-Aldrich) inhibitors, and 20 µM PMSF in isopropanol (P7626, Sigma-Aldrich). All experimental replicates were performed independently.

#### Chemical inhibitors used in cyclical stretch system

When used, inhibitor compounds were applied in DMEM supplemented with 1% FCS and 4 mM L-glutamine 30 min before stretching commenced. The activin receptor-like kinase (ALK5)/type I TGFβ-receptor kinase inhibitor SB-525334 (S8822, Sigma-Aldrich) was used at a concentration of 50 µM. The ROCK inhibitor Y27632 (Y0503, Sigma-Aldrich), the pan-αv integrin inhibitor CWHM-12 [a gift from Dr David Griggs (St Louis University, MI, USA)], the β1 integrin inhibitor NOTT199SS [a gift from Dr Thomas McInally (University of Nottingham, UK)], the MMP inhibitor GM6001 (CC1010, Sigma) and the serine protease inhibitor AEBSF (SBR00015, Sigma-Aldrich) were used at varying concentrations. Where inhibitors were dissolved in DMSO, the negative-control cells were treated with a DMSO concentration equivalent to that used in the highest inhibitor concentration.

#### GNAQ and GNA11 siRNA

siRNAs for human *GNAQ* (ON-TARGET-plus SMARTpool GNAQ, L-008562-00-0005, Dharmacon) and *GNA11* (ON-TARGET-plus SMARTpool GNA11, L-010860-00-0005, Dharmacon) were used to induce *GNAQ* and *GNA11* knockdown. A non-targeting siRNA pool was used as a control (ON-TARGET-plus non-targeting pool, D-001810-10-05, Dharmacon).

Cells were seeded at 1.5×10^5^ cells per well of collagen I-coated Bioflex^®^ 6-well culture plates (3001-C, Dunn Labortechnik) in antibiotic-free DMEM supplemented with 10% FCS and 4 mM L-glutamine. The following day, *GNAQ* and *GNA11* siRNA was applied at a concentration of 15 nM each with 4 µl/ml DharmaFECT 1 transfection reagent (T-2001-01, Dharmacon) as per the manufacturer's protocol. At 48 h after transfection, the media was changed to DMEM supplemented with 1% FCS and 4 mM L-glutamine. Cyclical stretch was applied for 24 h from 72 h post-transfection. G_αq/11_ knockdown was confirmed by western blotting and qPCR.

### Western blotting

Protein concentrations were determined by BCA assay using a commercially available kit (PN23227, Thermo Fisher Scientific), according to the manufacturer's instructions. Equal amounts of protein (15-25 µg) were loaded per lane of a 10% SDS-polyacrylamide gel and subjected to electrophoresis, then transferred onto a polyvinylidene fluoride membrane (1620177, Bio-Rad). Membranes were blocked for 1 h in either 5% non-fat milk (pSmad2, Smad2/3, αSMA, G_αq_, G_α11_, GAPDH) or 3% bovine serum albumin (TGFβ1, TGFβ2) in Tris-buffered saline containing 0.1% Tween 20, pH 7.4 (TBST). Membranes were incubated overnight at 4°C in blocking buffer with the appropriate primary antibody. Membranes were washed in TBST, then incubated for 1-2 h in the appropriate horseradish peroxidase (HRP)-conjugated secondary antibody in blocking buffer. Western blots were analysed using chemilluminescence and exposure to film (28-9068-35, GE Healthcare). Where membranes were probed for two different proteins of the same molecular weight, i.e. pSmad2 and Smad2, the membrane was stripped after analysis of pSmad2 using Western Restore Stripping Buffer (21059, Thermo Fisher Scientific) for 5 min and re-blocked with 5% non-fat milk before application of the second primary antibody.

The following antibodies were used for western blots: rabbit anti-phospho-Smad2 (pSmad2) (Cell Signaling Technology, 3108; 1:1000), rabbit anti-Smad2/3 (Cell Signaling Technology, 3102; 1:1000), rabbit anti-αSMA (Abcam, ab5694; 0.5 µg/ml), rabbit anti-GAPDH (Abcam, ab181603; 1:10,000), rabbit anti-TGFβ1 (ab92486; 4 µg/ml), mouse anti-TGFβ2 (Abcam, ab36495; 1:1000), rabbit anti G_α11_ (Abcam, ab153951; 1:1000), goat anti-G_αq_ (Abcam, ab128060; 0.1 µg/ml), HRP-conjugated goat anti-rabbit (Agilent, P044801-2; 1:3000), HRP-conjugated rabbit anti-goat (Agilent, P016002-2; 1:3000), HRP-conjugated rabbit anti-mouse (Agilent, P0260022-2, 1:3000).

#### Densitometry analysis of western blots

Densitometry was performed using ImageJ on scanned western blot images. JPEG images were converted into greyscale images, and the software used to calculate densitometry values for each band relative to the other bands. These relative densitometry values were used to calculate the expression of protein relative to loading control using the equation: protein relative to loading control=protein densitometry value/loading control protein densitometry value.

### Quantitative PCR

RNA was isolated from *in vitro* experiments using the Machery-Nagel Nucleospin RNA isolation kit (740955.250) according to the manufacturer's instructions. Complementary DNA (cDNA) was reverse transcribed from 200 µg RNA using Superscript IV Reverse Transcriptase (18090050, Thermo Fisher Scientific) according to the manufacturer's protocol. Quantitative PCR was performed on cDNA using gene-specific primers (see below), and an MXPro3000 qPCR machine (Stratagene) at an annealing temperature of 60°C for 40 cycles. KAPA SYBR FastTaq (KK4618, Sigma-Aldrich) was used for qPCR of all genes other than *Pdgfa*, *Pdgfb*, *Pdgfc*, *Pdgfd*, *Pdgfra* and *Pdgfrb*, for which PerfeCTa SYBR Green Fastmix (733-1382, VWR) was used. Amplification of a single PCR product was confirmed by melting curve analysis. The ΔΔCt method was used to quantify gene expression relative to the housekeeping genes *Hprt* (mouse samples) or *B2M* (human samples).

Primers were obtained from Eurofins. Sequences for mouse genes were: *Hprt* forward 5′-TGAAAGACTTGCTCGAGATGTCA-3′, *Hprt* reverse 5′-CCAGCAGGTCAGCAAAGAACT-3′; *Acta2* forward 5′-GGGATCCTGACGCTGAAGTA-3′, *Acta2* reverse 5′-GACAGCACAGCCTGAATAGC-3′; *Eln* forward 5′ GATGGTGCACACCTTTGTTG-3′, *Eln* reverse 5′-CAGTGTGAGCCATCTCA-3′; *Col1a1* forward 5′-AGCTTTGTGCACCTCCGGCT-3′, *Col1a1* reverse 5′-ACACAGCCGTGCCATTGTGG-3′; *Col3a1* forward 5′-TTTGCAGCCTGGGCTCATTT-3′, *Col3a1* reverse 5′-AGGTACCGATTTGAACAGACT-3′; *Pdgfa* forward 5′-GAGATACCCCGGGAGTTGA-3′, *Pdgfa* reverse 5′-TCTTGCAAACTGCAGGAATG-3′; *Pdgfb* forward 5′-TGAAATGCTGAGCGACCAC-3′, *Pdgfb* reverse 5′-AGCTTTCCAACTCGACTCC-3′; *Pdgfc* forward 5′-AGGTTGTCTCCTGGTCAAGC-3′, *Pdgfc* reverse 5′-CCTGCGTTTCCTCTACACAC-3′; *Pdgfd* forward 5′-CCAAGGAACCTGCTTCTGAC-3′, *Pdgfd* reverse 5′-CTTGGAGGGATCTCCTTGTG-3′; *Pdgfra* forward 5′-CAAACCCTGAGACCACAATG-3′, *Pdgfra* reverse 5′-TCCCCCAACAGTAACCCAAG-3′; *Pdgfrb* forward 5′-TGCCTCAGCCAAATGTCACC-3′, *Pdgfrb* reverse 5′-TGCTCACCACCTCGTATTCC-3′.

Primer sequences for human genes were: *GNAQ* forward 5′-GGACAGGAGAGGGTGGCAAG-3′, *GNAQ* reverse 5′-TGGGATCTTGAGTGTGTCCA-3′; *GNA11* forward 5′-CCACTGCTTTGAGAACGTGA-3′, *GNA11* reverse 5′-GCAGGTCCTTCTTGTTGAGG-3′; *B2M* forward 5′-AATCCAAATGCGGCATCT-3′, *B2M* reverse 5′-GAGTATGCCTGCCGTGTG-3′.

### Statistical analyses

Statistical analyses were performed using GraphPad Prism 8.2 software. For experiments with group sizes of ≤5, a non-parametric test was used. For experiments with group sizes of >5, data were assessed for normality and a parametric test used if data followed a normal distribution.

## Supplementary Material

10.1242/develop.201046_sup1 Supplementary information
