# Supplementary material for: Stretch regulates alveologenesis and homeostasis via mesenchymal Gαq/11-mediated TGFβ2 activation
Source: Development. 2023 May 12;150(9):dev201046. doi: 10.1242/dev.201046 (PMC10259661; doi:10.1242/dev.201046)
Supplement: Supplementary information [file develop-150-201046-s1.pdf]

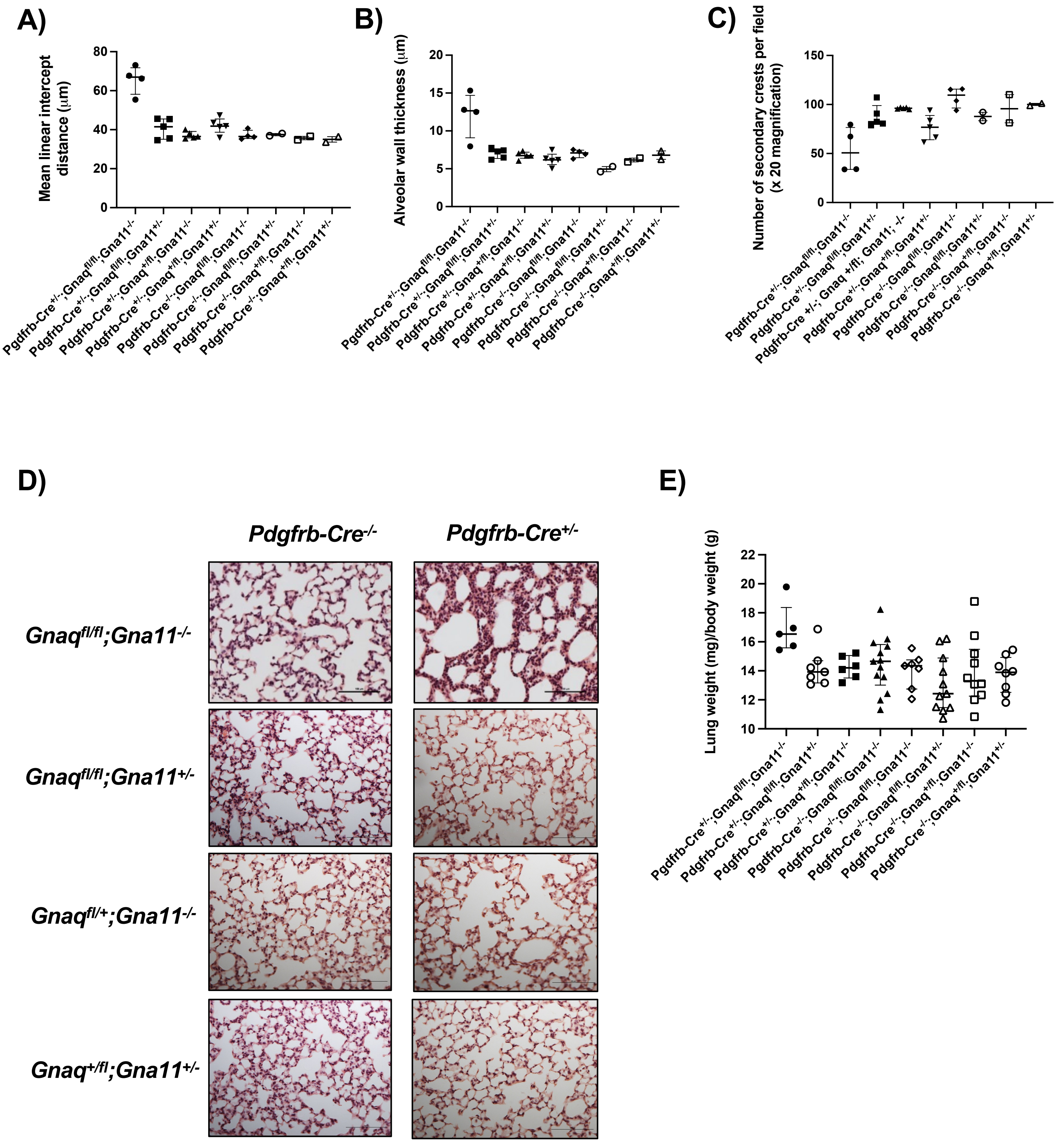

Development • Supplementary information

Fig. S1. Mice with at least one functioning *Gnaq* or *Gna11* allele have normal lung morphology

A) Mean linear intercept measurements from mice of all possible genotypes from the *Pdgfrb-Cre*$^{+/-}$ x *Gnaq*$^{fl/fl}$;*Gna11*$^{-/-}$ breeding programme. Median ± interquartile range, n=2-5 mice per genotype.

B) Alveolar wall thickness measurements from mice of all possible genotypes from the *Pdgfrb-Cre*$^{+/-}$ x *Gnaq*$^{fl/fl}$;*Gna11*$^{-/-}$ breeding programme. Median ± interquartile range, n=2-5 mice per genotype.

C) Quantification of secondary crests from mice of all possible genotypes from the *Pdgfrb-Cre*$^{+/-}$ x *Gnaq*$^{fl/fl}$;*Gna11*$^{-/-}$ breeding programme. Median ± interquartile range, n=2-5 mice per genotype.

D) Representative histology images from mice of all possible genotypes from the *Pdgfrb-Cre*$^{+/-}$ x *Gnaq*$^{fl/fl}$;*Gna11*$^{-/-}$ breeding programme.

E) Relative lung weight (mg) corrected to total body weight (g) of mice of all possible genotypes from the *Pdgfrb-Cre*$^{+/-}$ x *Gnaq*$^{fl/fl}$;*Gna11*$^{-/-}$ breeding programme. Median ± interquartile range, n=5-13 mice per genotype.

Development • Supplementary information

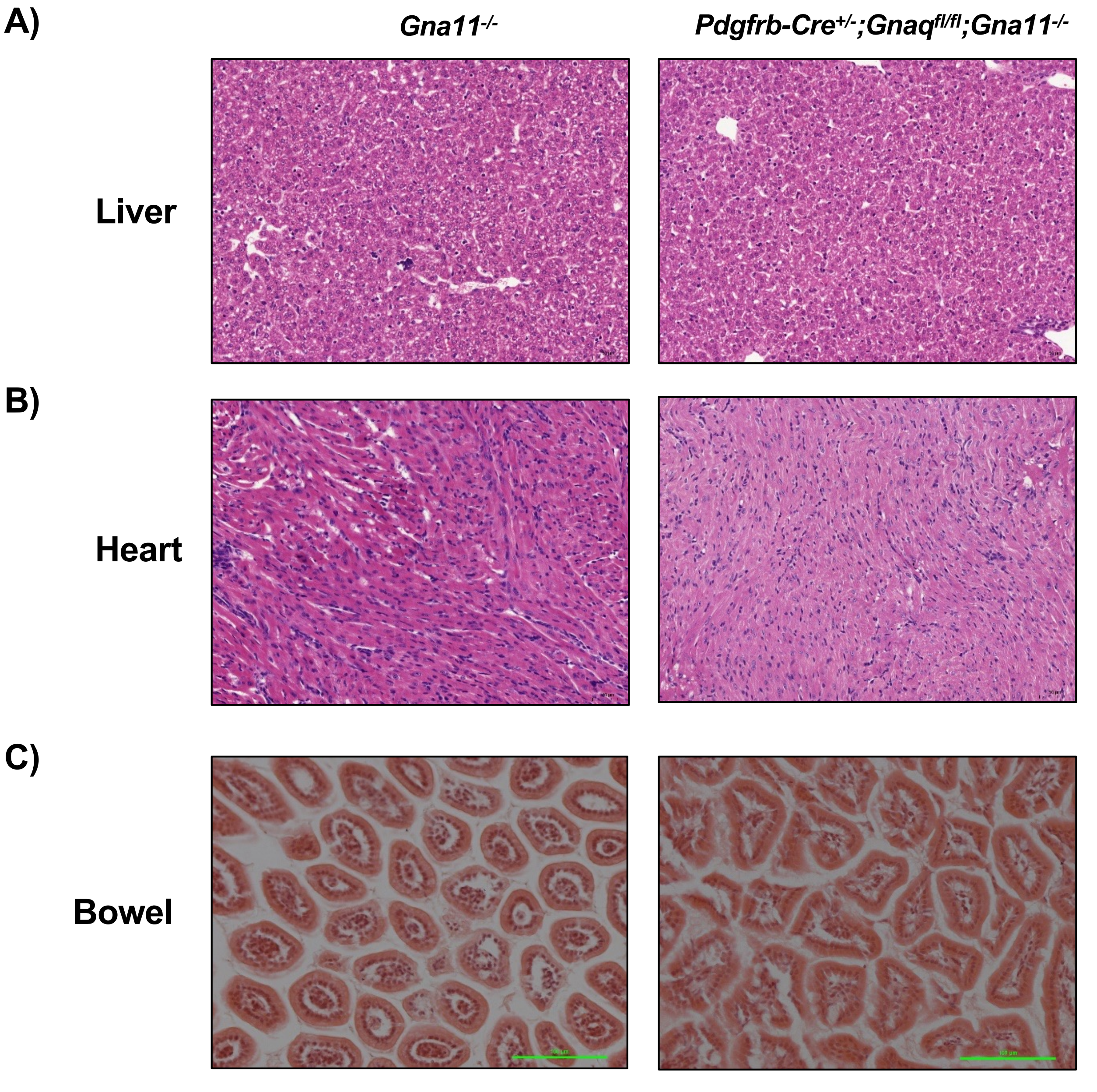

Fig. S2. ***Pdgfrb-Cre$^{+/-}$;Gnaq$^{fl/fl}$;Gna11$^{-/-}$*** **mice have normal liver, heart, and bowel histology.**

Representative images of haematoxylin and eosin staining of liver (A), heart (B), and bowel (C) from *Gna11$^{-/-}$* and *Pdgfrb-Cre$^{+/-}$;Gnaq$^{fl/fl}$;Gna11$^{-/-}$* mice.

Gna11$^{-/-}$ = *Pdgfrb-Cre$^{-/-}$;Gnaq$^{fl/fl}$;Gna11$^{-/-}$* littermate controls

Development • Supplementary information

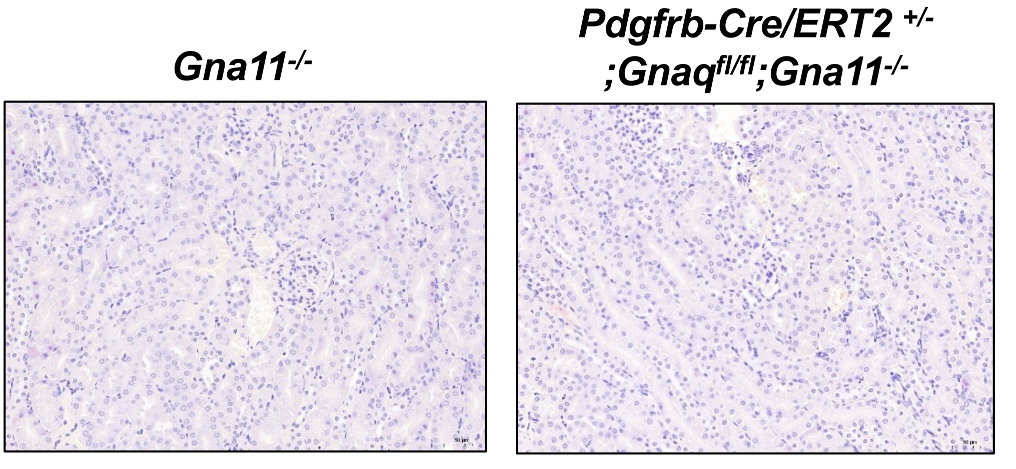

Fig. S3. ***Pdgfrb-Cre/ERT2$^{+/-}$;Gnaq$^{fl/fl}$;Gna11$^{-/-}$*** **mice have normal kidney histology after three weeks of tamoxifen.**

Representative images of haematoxylin and eosin staining of kidney from *Gna11$^{-/-}$* and *Pdgfrb-Cre/ERT2$^{+/-}$;Gnaq$^{fl/fl}$;Gna11$^{-/-}$* mice treated with three weeks of tamoxifen.

Gna11$^{-/-}$ = *Pdgfrb-Cre/ERT2$^{-/-}$;Gnaq$^{fl/fl}$;Gna11$^{-/-}$* littermate controls

Development • Supplementary information

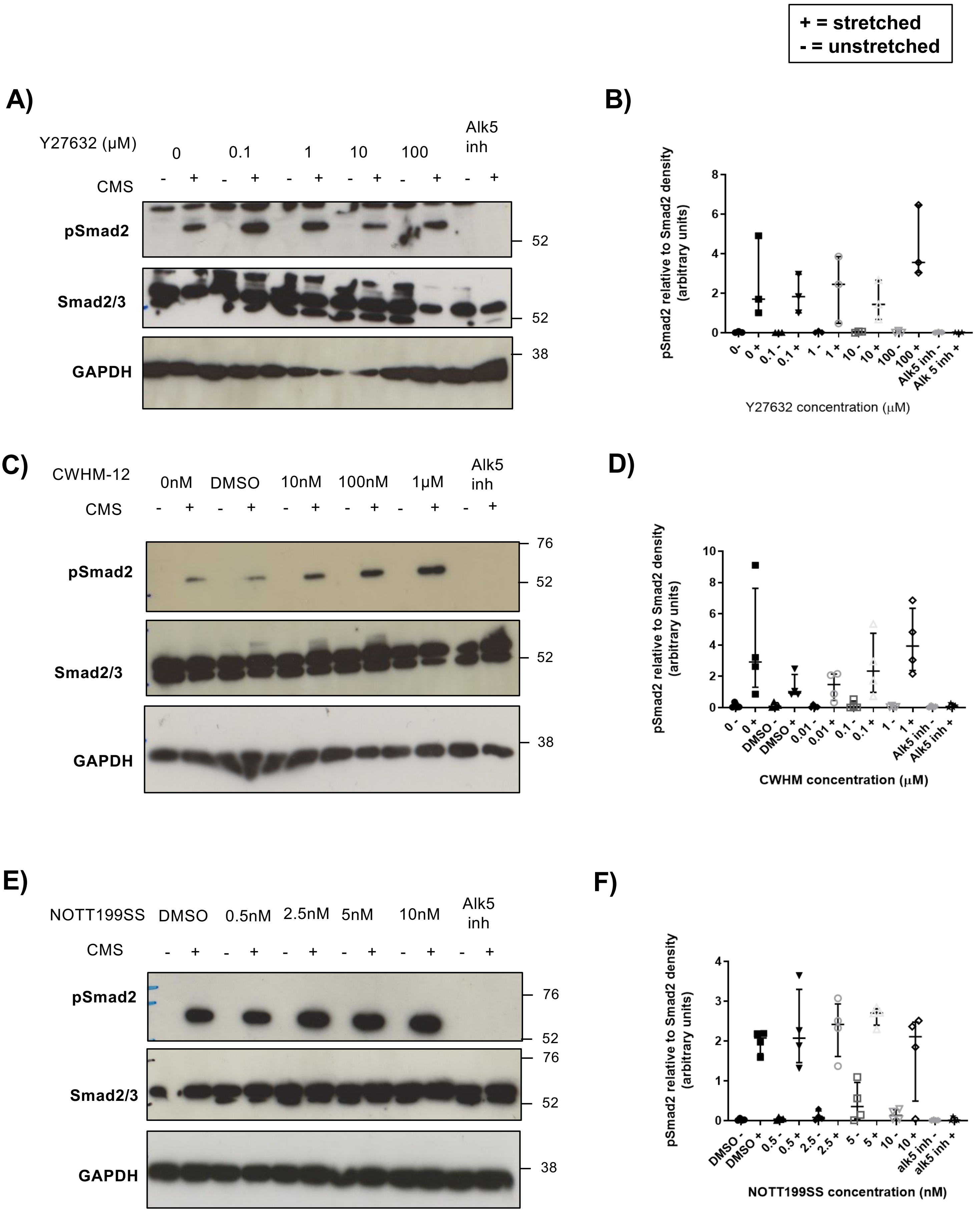

Development • Supplementary information

Fig. S4. Cyclical stretch-induced TGFβ activation occurs independently of ROCK, and αv and β1 integrins in fibroblasts.

A) Representative pSmad2 western blot of human lung fibroblasts treated with the ROCK inhibitor Y27632 then subject to 48 hours of cyclical mechanical stretch (CMS) (15% elongation, 0.3Hz, 48 hours).

B) Relative pSmad2 to Smad2 densitometry from western blots of human lung fibroblasts treated with Y27632 then subject to cyclical stretch. Median ± interquartile range, n=4, two-tailed Mann Whitney test.

C) Representative pSmad2 western blot of human lung fibroblasts treated with an αv integrin inhibitor (CWHM-12) then subject to 48 hours of cyclical stretch (15% elongation, 0.3Hz, 48 hours).

D) Relative pSmad2 to Smad2 densitometry of human lung fibroblasts treated with CWHM-12 then subject to cyclical stretch. Median ± interquartile range, n=4, two-tailed Mann Whitney test.

E) Representative pSmad2 western blot of human lung fibroblasts treated with a β1 integrin inhibitor (NOTT199SS) then subject to 48 hours of cyclical stretch (15% elongation, 0.3Hz, 48 hours).

F) pSmad2 relative to Smad2 densitometry of human lung fibroblasts treated with NOTT199SS then subject to cyclical stretch. Median ± interquartile range, n=4, two-tailed Mann Whitney Test.

- + = stretched; - = unstretched; CMS= cyclical mechanical stretch; Y27632 = ROCK inhibitor; CWHM-12 = pan αv integrin inhibitor; NOTT199SS = β1 integrin inhibitor; DMSO = dimethyl sulfoxide; Alk5 inh = 50μM Alk5 inhibitor (SB525334)

Development • Supplementary information

Supp figure 5

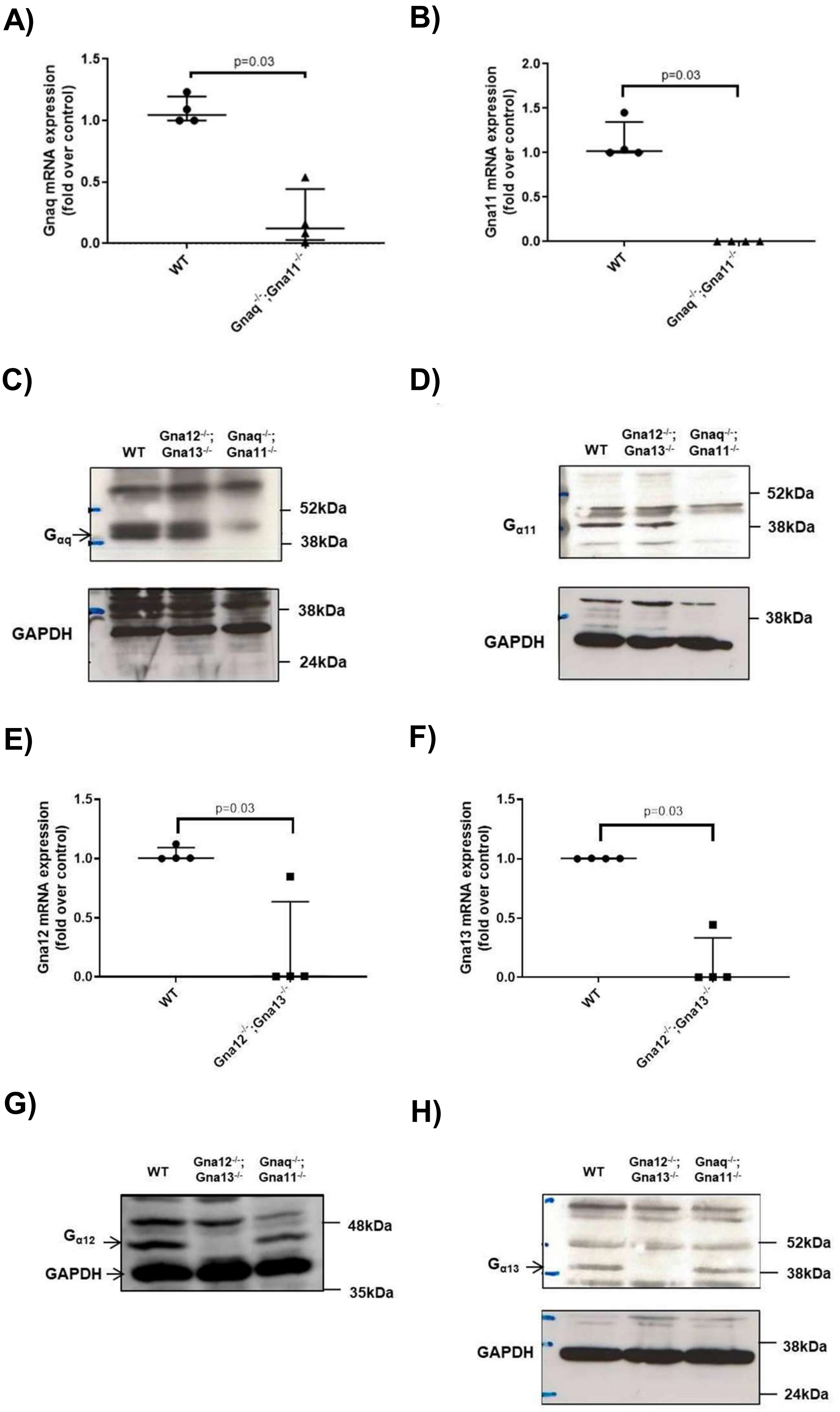

Development • Supplementary information

Fig. S5. $G_{\alpha q/11}$ and $G_{\alpha 12/13}$ knockdown is confirmed in *Gnaq$^{-/-}$;Gna11$^{-/-}$* and *Gna12$^{-/-}$;Gna13$^{-/-}$* MEFs, respectively.

A) *Gnaq* mRNA expression in wild-type (WT) and Gnaq$^{-/-}$;Gna11$^{-/-}$ MEFs. Median ± interquartile range, n=4, two-tailed Mann Whitney Test.

B) *Gna11* mRNA expression in wild-type (WT) and Gnaq$^{-/-}$;Gna11$^{-/-}$ MEFs. Median ± interquartile range, n=4, two-tailed Mann Whitney Test.

C) Representative Gαq western blot on wild-type (WT), *Gnaq$^{-/-}$;Gna11$^{-/-}$* and *Gna12$^{-/-}$;Gna13$^{-/-}$* MEFs. Western blots are representative of three independent experiments.

D) Representative Gα11 western blot on wild-type (WT), *Gnaq$^{-/-}$;Gna11$^{-/-}$* and *Gna12$^{-/-}$;Gna13$^{-/-}$* MEFs. Western blots are representative of three independent experiments.

E) *Gna12* mRNA expression in wild-type (WT) and *Gna12$^{-/-}$;Gna13$^{-/-}$* MEFs. Median ± interquartile range, n=4, two-tailed Mann Whitney Test.

F) *Gna13* mRNA expression in wild-type (WT) and *Gna12$^{-/-}$;Gna13$^{-/-}$* MEFs. Median ± interquartile range, n=4, two-tailed Mann Whitney Test.

G) Representative Gα12 western blot on wild-type (WT), *Gnaq$^{-/-}$;Gna11$^{-/-}$* and *Gna12$^{-/-}$;Gna13$^{-/-}$* MEFs. Western blots are representative of three independent experiments.

H) Representative Gα13 western blot on wild-type (WT), *Gnaq$^{-/-}$;Gna11$^{-/-}$* and *Gna12$^{-/-}$;Gna13$^{-/-}$* MEFs. Western blots are representative of three independent experiments.

Development • Supplementary information

Table S1. Resources and reagents used for this work

REAGENT or RESOURCE	SOURCE	IDENTIFIER
Antibodies		
Rabbit anti-phospho-Smad2 (pSmad2)	Cell Signaling Technology	Cat# 3808L
Rabbit anti-Smad2/3	Cell Signaling Technology	Cat# 3102
Rabbit anti-α-smooth muscle actin (αSMA)	Abcam	Cat# ab5694
Rabbit anti-GAPDH	Abcam	Cat# ab181603
Rabbit anti-TGFβ1	Abcam	Cat# ab92486
Rabbit anti-elastin	Atlas	Cat # HPA056941
Mouse anti-TGFβ2	Abcam	Cat# ab36495
Rabbit anti $G_{\alpha 11}$	Abcam	Cat# ab153951
Goat anti-$G_{\alpha q}$	Abcam	Cat# ab128060
HRP-conjugated goat-anti-rabbit	Agilent	Cat# P044801-2
HRP-conjugated rabbit-anti-goat	Agilent	Cat# P016002-2
HRP-conjugated rabbit anti-mouse	Agilent	Cat# P0260022-2
Rabbit anti-CD31	Abcam	Cat# ab182981
Rabbit anti-ki67	Abcam	Cat# ab15580
Rabbit anti-pro-surfactant protein C	Sigma	Cat# Ab3786
Rabbit anti-TGFβ2	Proteintech	Cat# 19999-1-AP
Biotinylated goat anti-rabbit IgG	Vector	Cat# BA1000
Chemicals, Peptides, and Recombinant Proteins		
Protein lysis buffer	Cell Signaling Technology	Cat# 9803
Phos-stop phosphatase inhibitors	Sigma	Cat# 04906837001
Complete mini protease inhibitors	Sigma	Cat# 04693124001
PMSF	Sigma	Cat# P7626
SB-525334 (ALK5 inhibitor)	Sigma	Cat# S8822

Development • Supplementary information

Y27632 (ROCK inhibitor)	Sigma	Cat# Y0503
CWHM-12 (αv integrin inhibitor)	A gift from Dr David Griggs, University of St Louis. Now commercially available from various suppliers	https://www.medchemexpress.com/CWHM-12.html<br><br>https://medkoo.com/products/11038<br><br>https://www.caymanchem.com/product/19480/cwhm12
NOTT199SS	School of Chemistry at the University of Nottingham	n/a
GM6001 (MMP inhibitor)	Sigma	Cat# CC1010
DharmaFECT 1 transfection reagent	Dharmacon	Cat# T-2001-01
10% formalin	VWR	Cat# 11699404
Mayers haematoxylin	Sigma	Cat# S1275
Eosin	VWR	Cat# 101411-524
Hydrogen peroxide	VWR	Cat# 23619.264
SIGMAFAST(TM) 3,3'-Diaminobenzidine tablets	Sigma	Cat# D4418
AEBSF (serine protease inhibitor	Sigma	Cat# SBR00015
Western Restore Stripping Buffer	Thermo-Fisher	Cat# 21059
Ferric chloride (Iron(III) chloride)	Sigma	Cat# 157740
Iodine	Sigma	Cat# 326143
Potassium iodide	Sigma	Cat# 03124
Picric acid (in aqueous solution)	VWR	Cat# 84512.260
Acid fuschin	Sigma	Cat# F8129

Development • Supplementary information

Direct red 80	Sigma	Cat# 365548
Sodium thiosulphate	Scientific Laboratory Supplies	Cat# 72049
Haematoxylin	Sigma	Cat# H3136
Experimental Models: Cell Lines		
Human lung fibroblasts – primary cultures	Isolated and cultured in house (see methods for details)	n/a
Murine embryonic fibroblasts – wild-type	(Gu et al. 2002; Zywietz et al. 2001)	n/a
Murine embryonic fibroblasts – $Gnaq^{-/-}Gna11^{-/-}$	(Gu et al. 2002; Zywietz et al. 2001)	n/a
Murine embryonic fibroblasts – $Gna12^{-/-}$;$Gna13^{-/-}$	(Gu et al. 2002; Zywietz et al. 2001)	n/a
Experimental Models: Organisms/Strains		
$Pdgfrb\text{-}Cre^{+/-}$ mice	Generation described in (Foo et al. 2006)	n/a
$Pdgfrb\text{-}Cre/ERT2^{+/-+}$ mice	Jackson Laboratories	Cat # 029684
$Gnaq^{fl/fl}$;$Gna11^{-/-}$ mice	Generation described in (Offermanns et al. 1998; Wettschureck et al. 2001). Sperm stored in lab of origin.	n/a
Oligonucleotides		

Development • Supplementary information

Genotyping primers: Cre recombinase<br>5'- GCG GTC TGG CAG TAA AAA CTA TC – 3';<br>5' - GTG AAA CAG CAT TGC TGT CAC TT – 3'	Eurofins (custom order)	n/a
Genotyping primers: internal positive control<br>5' - CTA GGC CAC AGA ATT GAA AGA TCT – 3'<br>5' - GTA GGT GGA AAT TCT AGC ATC C – 3'	Eurofins (custom order)	n/a
Genotyping primers: Gna11 wild type<br>5' – AGC ATG CTG TAA GAC CGT AG - 3'<br>5' – GCC CCT TGT ACA GAT GGC AG – 3'	Eurofins (custom order)	n/a
Genotyping primers: Gna11 knockout<br>5' - CAG GGG TAG GTG ATG ATT GTG – 3'<br>5' – GAC TAG TGA GAC GTG CTA CTT CC - 3'	Eurofins (custom order)	n/a
Genotyping primers: Gnaq<br>5' – GCA TGC GTG TCC TTT ATG TGA G 3'<br>5' – AGC TTA GTC TGG TGA CAG AAG – 3'	Eurofins (custom order)	n/a
Genotypring primers: Cre/ERT2<br>5'- GAA CTG TCA CCG GGA - 3'<br>5' - AGG CAA ATT TTG GTG TAC GG – 3'	Eurofins (custom order)	n/a
Human GNAQ siRNA (ON-TARGET-plus SMARTpool)	Dharmacon	Cat# L-008562-00-0005
Human GNA11 siRNA (ON-TARGET-plus SMARTpool)	Dharmacon	Cat# L-010860-00-0005
Non-targeting siRNA pool (ON-TARGET-plus SMARTpool)	Dharmacon	Cat# D-001810-10-05
Mouse Hprt primer forward: 5' – TGA AAG ACT TGC TCG AGA TGT CA - 3'	Eurofins (custom order)	n/a
Mouse Hprt primer reverse: 5' – CCA GCA GGT CAG CAA AGA ACT 3'	Eurofins (custom order)	n/a

Development • Supplementary information

Mouse Acta2 primer forward: 5’ - GGG ATC CTG ACG CTG AAG TA – 3’	Eurofins (custom order)	n/a
Mouse Acta2 primer reverse: 5’ – GAC AGC ACA GCC TGA ATA GC – 3’	Eurofins (custom order)	n/a
Mouse Eln primer forward: 5' GAT GGT GCA CAC CTT TGT TG 3'	Eurofins (custom order)	n/a
Mouse Eln primer reverse: 5' CAG TGT GAG CCA TCT CA 3'	Eurofins (custom order)	n/a
Mouse Col1a1 primer forward: 5' AGC TTT GTG CAC CTC CGG CT 3'	Eurofins (custom order)	n/a
Mouse Col1a1 primer reverse: 5' ACA CAG CCG TGC CAT TGT GG 3'	Eurofins (custom order)	n/a
Mouse Col3a1 primer forward: 5' TTT GCA GCC TGG GCT CAT TT 3'	Eurofins (custom order)	n/a
Mouse Col3a1 primer reverse: 5' AGG TAC CGA TTT GAA CAG ACT 3’	Eurofins (custom order)	n/a
Mouse *Pdgfa* primer forward: 5’ GAG ATA CCC CGG GAG TTG A 3’	Eurofins (custom order)	n/a
Mouse *Pdgfa* primer reverse: 5’ TCT TGC AAA CTG CAG GAA TG 3’	Eurofins (custom order)	n/a
Mouse *Pdgfb* primer forward: 5’ TGA AAT GCT GAG CGA CCA C 3’	Eurofins (custom order)	n/a
Mouse *Pdgfb* primer reverse: 5’ AGC TTT CCA ACT CGA CTC C 3’	Eurofins (custom order)	n/a
Mouse *Pdgfc* primer forward: 5’ AGG TTG TCT CCT GGT CAA GC 3’	Eurofins (custom order)	n/a
Mouse *Pdgfc* primer reverse: 5’ CCT GCG TTT CCT CTA CAC AC 3’	Eurofins (custom order)	n/a
Mouse *Pdgfd* primer forward: 5’CCA AGG AAC CTG CTT CTG AC 3’	Eurofins (custom order)	n/a

Development • Supplementary information

Mouse *Pdgfd* primer reverse: 5’ CTT GGA GGG ATC TCC TTG TG 3’	Eurofins (custom order)	n/a
Mouse *Pdgfra* primer forward: 5’ CAA ACC CTG AGA CCA CAA TG 3’	Eurofins (custom order)	n/a
Mouse *Pdgfra* primer reverse: 5’ TCC CCC AAC AGT AAC CCA AG 3’	Eurofins (custom order)	n/a
Mouse *Pdgfrb* primer forward: TGC CTC AGC CAA ATG TCA CC 3’	Eurofins (custom order)	n/a
Mouse *Pdgfrb* primer reverse: 5’ TGC TCA CCA CCT CGT ATT CC 3’	Eurofins (custom order)	n/a
Human GNAQ primer forward: 5’ – GGACAGGAGAGGGTGGCAAG – 3’	Eurofins (custom order)	n/a
Human GNAQ primer reverse: 5’ – TGGGATCTTGAGTGTGTCCA – 3’	Eurofins (custom order)	n/a
Human GNA11 primer forward: 5’ – CCACTGCTTTGAGAACGTGA – 3’	Eurofins (custom order)	n/a
Human GNA11 primer reverse: 5’ GCAGGTCCTTCTTGTTGAGG – 3’	Eurofins (custom order)	n/a
Human B2M primer forward: 5’-AATCCAAATGCGGCATCT-3’	Eurofins (custom order)	n/a
Human B2M primer reverse: 5’-GAGTATGCCTGCCGTGTG-3’	Eurofins (custom order)	n/a
Software and Algorithms		
Image J	NIH	https://imagej.nih.gov/ij/
NIH Elements v3.2	Nikon	https://www.microscope.healthcare.nikon.com/products/software/nis-elements/viewer

MicroManager 1.4	Vale lab, UCSF	https://micro-manager.org/
CaseViewer 2.3	3D Histech	https://www.3dhistech.com/
Prism	Graphpad	https://www.graphpad.com/scientific-software/prism/
Other		
Goat serum	Sigma	Cat# G9023
Avidin-Biotin complex	Vector	Cat# SP2001
DPX mountant	Sigma	Cat# 06522
Phosphate buffered saline	Sigma	Cat# P4417
Nikon 90i microscope	Nikon	n/a
Axioplan microscope	Zeiss	n/a
Dulbecco's modified eagles medium	Sigma	D5671
Foetal Calf Serum	Harlan UK Ltd	S-0001AE
L-glutamine	Sigma	G7513
Penicillin/ streptomycin	Sigma	P4458
Amphotericin B	Sigma	Cat# A2942
Collagen I-coated Bioflex® 6 well culture plates	Dunn Labortechnik	Cat# 3001-C
Flexcell® cell stretching system	Flexcell International Corporation	Cat# FX-5000T
BCA Assay kit	ThermoFisher	Cat# PN23227
Nucleospin RNA isolation kit	Machery-Nagel	Cat# 740955.250
Superscript IV Reverse Transcriptase	ThermoFisher	Cat# 18090050
KAPA SYBR FastTaq	Sigma	Cat# KK4618
PerfeCTa® SYBR Green Fastmix	VWR	Cat# 733-1382
MXPro3000 qPCR machine	Stratagene	

Development • Supplementary information

Heparin sodium 5000 units/ml	Wockhardt	Cat# FP1083
Polyvinylidene fluoride membrane	BioRad	Cat# 1620177
Hyperfilm for western blots	GE Healthcare	Cat# 28-9068-35
ECL reagent	GE Healthcare	Cat# RPN2134
Clarity ECL	BioRad	Cat# **1705061**
Tamoxifen-containing chow (400mg/kg tamoxifen citrate)	Envigo	Cat# TD.55125.1
