# Supplementary material for: Stretch regulates alveologenesis and homeostasis via mesenchymal Gαq/11-mediated TGFβ2 activation
Source: Development. 2023 May 12;150(9):dev201046. doi: 10.1242/dev.201046 (PMC10259661; doi:10.1242/dev.201046)
Supplement: Supplementary file 1 [file develop-150-201046_review_history.pdf]

Stretch regulates alveologenesis and homeostasis via mesenchymal $G_{\alpha q/11}$-mediated TGFβ2 activation

Amanda T. Goodwin, Alison E. John, Chitra Joseph, Anthony Habgood, Amanda L. Tatler, Katalin Susztak, Matthew Palmer, Stefan Offermanns, Neil C. Henderson and R. Gisli Jenkins

DOI: 10.1242/dev.201046

Editor: Liz Robertson

Review timeline

Original submission:	27 Sep 2020
Editorial decision:	26 Nov 2020
Resubmission:	4 Jul 2022
Editorial decision:	23 Aug 2023
First revision:	1 Mar 2023
Accepted:	5 Apr 2023

Original submission

First decision letter

MS ID#: DEVELOP/2020/197368

MS TITLE: Stretch Regulates Alveologenesis Via Mesenchymal $G_{\alpha q/11}$-mediated TGFβ2 Activation

AUTHORS: Amanda T Goodwin, Alison E John, Chitra Joseph, Anthony Habgood, Amanda L Tatler, Stefan Offermanns, Neil C Henderson, and Gisli Jenkins

ARTICLE TYPE: Research Article

Dear Dr. Goodwin,

Many apologies for the delay in getting back to you about your manuscript. I have now received all the referees reports and have reached a decision. The referees' comments are appended below, or you can access them online: please go to BenchPress and click on the 'Manuscripts with Decisions' queue in the Author Area.

As you will see from their reports, the referees recognise the potential of your work, but they also raise significant concerns about it. These are clearly articulated in the comments by Reviewer 2. Given the nature of these concerns, I am afraid I have little choice other than to reject the paper at this stage.

However, having evaluated the paper, I do recognise the potential importance of this work. I would therefore be prepared to consider as a new submission an extension of this study that contains new experiments, data and discussions and that address fully the major concerns of the referees. The work required goes beyond a standard revision of the paper. Please bear in mind that the referees (who may be different from the present reviewers) will assess the novelty of your work in the context of all previous publications, including those published between now and the time of resubmission.

© 2023. Published by The Company of Biologists under the terms of the Creative Commons Attribution License (https://creativecommons.org/licenses/by/4.0/).

Reviewer 1

Advance summary and potential significance to field

This paper shows strong evidence that alveolarization is strongly inhibited in P14 mice with a mesenchyme lineage specific knockout of certain Galpha2/11 proteins driven by the Pdgrb-Cre transgene into mesenchyme.
They further show that stretching lung fibroblasts increases the lrelaease of TGFbeta2 and that this is inhibited in cells with these G proteins knocked out. They therefore speculate that TGFbeta2 is the downstream target of stretch and that this induction and activation occurs a unique pathway via these particular G-proteins.
This is an important advance for students of alveolarization.

Comments for the author

The paper is well written and the work appears to have been done carefully.
There are a few spelling mistakes within the figure labels.

One thing of interest that is under appreciated in this paper is the drastic reduction in elastin deposition within the alveolar walls, there being little to no elastin shown on the histologic staining in the knockout. This is important because correct deposition of elastin is essential for normal alveogenesis. So far it has been well established that PDGF signaling is involvedl upstream in this process. However the data in this paper suggests that TGFbeta2 and these G-proteins may also be important controllers of elastin deposition.

Another point is that the surviving knockout mice are severely dwarfed. Both starvation and dwarfism as well as air hunger can be enough to induce or be caused by hypoalveolarization and whether this is due to impaired gas exchange or increased metabolism might bear some thought. Pdgfr-Cre is also expressed elsewhere in the body and so probably are the G-proteins. So this is by no means a lung specific knockout system? Is their gut abnormal?

The suggestion that this discovery may be revelatory for lung cancer etc may be a bit of a reach?

Reviewer 2

Advance summary and potential significance to field

This paper addresses the role of mechanical forces and TGFbeta in the postnatal formation of the alveoli of the mouse lung. This is a really critical stage of lung development when the primitive saccules formed at the distal end of the lung buds before birth are subdivided into multiple smaller units. This process involves the formation of so-called "secondary septal ridges or crests" that contain contractile myofibroblasts expressing high levels of smooth muscle actin and depositing elastin fibers. Cells with the secondary crest myofibroblast phenotype are only present transiently but precisely how they initially arise, differentiate, and subdivide the sacs and sculpt the alveoli are all very important questions. Another important question is how TGFbeta sigaling fits into these complex processes.
The authors use conditional deletion of the genes encoding G protein ? subunits G?q and G?11 (G?q/G?11) to test their role in transmitting the mechanical stretch caused by breathing. They use a Pdgfrb-Cre driver to theoretically delete the genes in pericytes in the embryonic lung. Unfortunately, the authors seem to be unaware of the considerable (and still unresolved) complexity in mesenchymal lineages of the developing lung and recent work that has addressed the origin of the cells that can be called, for simplicity, secondary crest myofibroblasts (SCMFs). Key arguments in the paper are that Pdgfrb-Cre is driving recombination specifically in pericytes and that pericytes are the origin of SCMFs. Unfortunately, neither of these suppositions completely holds up against recent studies. The authors really need to confirm their findings with another driver for pericytes and/or find a way to induce recombination in Pdgfrb+ cells at a specific stage of development to exploit their interesting use of the floxed (G?q/G?11) alleles.

© 2023. Published by The Company of Biologists under the terms of the Creative Commons Attribution License (https://creativecommons.org/licenses/by/4.0/).

Comments for the author

1. As the authors themselves point out, a number of single cell analyses have now been done on mesenchymal cells from the mouse (and human) lung. These show that while Pdgfrb is certainly highly enriched in pericytes in the adult lung, other cells express the gene at lower levels. Since the authors are using a Cre allele rather than a CreEr allele they would have to go back and show precisely where recombination is taking place. It is possible they may find a progenitor cell that gives rise to both pericytes and SCMFs. Studies by Liu et al doi.org/10.1101/2020.07.15.203141 argue against this. On the other hand, the review by Riccetti et al doi.org/10.1016/j.matbio.2020.05.002 suggests that Pdgfrb is expressed in early precursors of airway smooth muscle (which may, in turn give rise to SCMFs).

2. More significantly, Li et al doi.org/10.7554/eLife.36865.001 have shown by lineage tracing that Pdgfra+ cells give rise to SCMFs. While it is still possible that some SCMFs are derived from Pdgfrb+ precursors, the work of Li et al needs to be acknowledged. To make their argument about pericytes as the source of SCMFs, the authors have to show that their method only targets pericytes and confirm by lineage tracing that pericytes give rise to SCMFs.

3. The authors should consider new ideas about how SCMFs subdivide the terminal saccules (Branchfield et al doi:10.1016/j.ydbio.2015.11.017), the role of contractile pathways in the SCMFs (Li et al 2020 doi: 10.1172/JCI132189) and new ideas about how mechanical forces affect the very earliest stages of alveolar cell differentiation, that begin at the canalicular stage of lung development (Li et al doi.org/10.1016/j.devcel.2018.01.008)

4. A key finding in this paper is that alveolar elastin is severely reduced in the double mutants. However, the staining is very difficult to see and the 3D disposition of the bundles hard to envisage. The authors should use the methods of visualizing elastin in the Branchfield et al and Li et al 2020 papers cited above.

5. The authors should consider more deeply the possibility that the alveolar defect is secondary to defects in the vascularization of the developing alveoli. 3D imaging with markers for endothelial cells might reveal important information. (Alternatively, as discussed above, their Cre driver may be giving recombination in early progenitors of smooth muscle which, in turn gives rise to SCMFs)

6. While the studies with cultured fibroblasts are interesting the main interest of this paper for the audience of development is the effect on lung development and this needs to be clarified.

Resubmission

First decision letter

MS ID#: DEVELOP/2022/201046

MS TITLE: Stretch Regulates Alveologenesis and Homeostasis Via Mesenchymal $G_{\alpha q/11}$-mediated TGFβ2 Activation

AUTHORS: Amanda T Goodwin, Alison E John, Chitra Joseph, Anthony Habgood, Amanda L Tatler, Katalin Susztak, Matthew Palmer, Stefan Offermanns, Neil C Henderson, and R Gisli Jenkins

I have now received all the referees' reports on the above manuscript, and have reached a decision. The referees' comments are appended below, or you can access them online: please go to BenchPress and click on the 'Manuscripts with Decisions' queue in the Author Area.

As you will see, the referees express considerable interest in your work, but have some significant criticisms and recommend a substantial revision of your manuscript before we can consider publication. If you are able to revise the manuscript along the lines suggested, which may involve

© 2023. Published by The Company of Biologists under the terms of the Creative Commons Attribution License (https://creativecommons.org/licenses/by/4.0/).

further experiments, I will be happy receive a revised version of the manuscript. Your revised paper will be re-reviewed by one or more of the original referees, and acceptance of your manuscript will depend on your addressing satisfactorily the reviewers' major concerns. In particular I hope you can make adjustments to the text in response to Reviewer 1's concerns about the origins of lung fibroblasts and also address the major points raised by Reviewer 3. Please also note that Development will normally permit only one round of major revision. If it would be helpful, you are welcome to contact us to discuss your revision in greater detail. Please send us a point-by-point response indicating your plans for addressing the referee's comments, and we will look over this and provide further guidance.

Please attend to all of the reviewers' comments and ensure that you clearly highlight all changes made in the revised manuscript. Please avoid using 'Tracked changes' in Word files as these are lost in PDF conversion. I should be grateful if you would also provide a point-by-point response detailing how you have dealt with the points raised by the reviewers in the 'Response to Reviewers' box. If you do not agree with any of their criticisms or suggestions please explain clearly why this is so.

Reviewer 1

Advance summary and potential significance to field

See original review

Comments for the author

The authors have made some changes to the text of their manuscript to address my comments. However, their discussion remains very confusing. In parts, there is some concession to the fact that Pdgfrb is not a specific marker of pericytes. Yet in other parts of the revised manuscript the authors persist in claiming that pericytes are progenitors of myofibroblasts. For example, in lines 449-455 they write "Furthermore, while we propose that abnormalities in pericyte differentiation and migration underlie the defective alveologenesis and emphysema in mesenchymal G aq/11 knockout mice, Pdgfrb is expressed by other cell types, including myofibroblasts, fibroblasts and vascular smooth muscle cells. While it is possible that disturbing TGFb signalling in these cell types contributed to the lung phenotype in mesenchymal G aq/11 knockout mice, pericytes are major progenitors for all these cell types, and are therefore likely to have played a primary role in the abnormalities observed".
Their claim that pericytes are "major progenitors for myofibroblasts and fibroblasts" is just not substantiated in this paper or elsewhere. Moreover, many of the studies are done with MEFs (mouse embryonic fibroblasts) and not with specific populations of mouse (or human) lung fibroblasts. It is just not adequate to talk about "lung fibroblasts" anymore. Without more specific analysis of fibroblast subpopulations and use of additional pericyte driver(s) this revised paper does not advance the field of lung development.

Minor comment
The authors state that they use Pdgfrb-Cre/ERT2+/- mice (Laboratories). Do they mean Jackson Laboratories? They need to cite the original generation of these mice.

Reviewer 2

Advance summary and potential significance to field

This paper makes the interesting point that G proteins mediating mesenchymal stretch and TGF beta activation are important for postnatal alveolarization in mice.

Comments for the author

The authors have done a decent job of responding to the reviewers comments given what they have already got in hand.
The lung phenotype in their mouse genetics models is certainly very striking and the idea that stretch might be driving alveolarization is novel and attractive and should be put out there for the field to evaluate.

© 2023. Published by The Company of Biologists under the terms of the Creative Commons Attribution License (https://creativecommons.org/licenses/by/4.0/).

I still think they are missing a trick by underplaying the significance of defective elastin deposition and distribution in their model. But I suppose that's up to them. Certainly elastin is stretchy and has a lot to do with force distribution and recoil in springy tissues like the lung.

Reviewer 3

Advance summary and potential significance to field

In their report, the authors address a topical and important aspect of lung development and homeostasis, namely how stretch may impact the development of the immature mouse lung, and the maintenance of the structure of the adult mouse lung.
In particular, the authors have identified mesenchymal Gaq/11 signaling as regulating myofibroblast function in a pathway involving the activation of TGFbeta2. This is a novel and exciting study. I have the following comments for the improvement of the manuscript:

Comments for the author

MAJOR COMMENTS:

1. Might the authors comment on how restricted genetic recombination directed by the Pdgfrb-Cre driver line? Is the cellular distribution of the recombination activity of the driver line restricted to the cell-types addressed in the report, in the developing lung? Might an mTmG panel be available for this time-point for the relevant mouse strains (perhaps this is already available from other strain characterisation reports, which could be referenced here).
Alternatively, can an additional short discussion be added to the manuscript to address this point?

2. Throughout, the authors have selected Pdgfrb-Cre-/-,Gnaqfl/fl,Gna11-/- mice for their control groups. This is understandable, given that the floxed Gnaq allele cannot be recombined in the absence of Cre. However, this control group does not permit consideration of two potentially important confounding variables in the control groups: the presence of a Gnaq floxed allele, and the lack of Cre. Whether the introduction of the floxed Gnaq allele has any impact on Gnaq expression in the absence of Cre, compared with mice carrying an unfloxed Gnaq allele is not known. Also, the impact of overexpression of Cre in the Pdgfrb-Cre-/+ “controls” is also not known, Do the authors have any information about how lung structure looks, or Gnaq is expressed, comparing their Pdgfrb-Cre-/-,Gnaqfl/fl,Gna11-/- control strain with a Pdgfrb-Cre-/+,Gnaq+/+,Gna11-/- strain? In not, might the strain selection be rationalized in the text?

3. In the Pdgfrb-Cre+/-,Gnaqfl/fl,Gna11-/- mice, lungs were considered heavier at P14, compared to Gna11+/+ mice. Two questions: was the control comparison made with Gna11+/+ mice, or with Pdgfrb-Cre+/- mice, or with Gna+/+ as stated (p. 5). (i) In the case of the latter, might the presence of Pdgfrb-Cre+/- with an unexpected off-target effect have contributed to this phenomenon in the experimental group, and (ii) irrespective of the Pdgfrb-Cre status of the control group, could this increased weight be due to fluid accumulation in the lungs (i.e. was alveolar or interstitial oedema present?).

4. Given the emphasis on pericytes in the text, the consideration of the lung vascular structure is well-taken (primarily presented in Fig. 4). Along these lines: the consideration of lung vascular structure rests on interpretation of immunohistochemical data, primarily, the estimation of maximum and minimum wall thickness (Fig. 4 A,J) and visual inspection of the cardiac septum (Fig. 4K), leading the authors to conclude that lung vessels were markedly abnormal (p. 6) and that cardiac septal wall thickness is unchanged, which the authors apparently took to mean that there was no evidence of pulmonary hypertension.
Additionally, the capillaries were considered to be normal in “appearance”. I find this data set in the manuscript to very thinly support the conclusions drawn from the data. (i) There are well-established technique to study lung (indeed, any) vascular structure, and in terms of lung pathology, standard morphometric techniques to study vascular wall thickness have not been employed.
Can the author provide other evidence of change sin lung conducting vessel structure. The immunohistochemically images provide little clarity on this point. Data in Figs.4H,I suggest increased minimum and maximum vessel wall thickness. Are we talking about the vascular media?

© 2023. Published by The Company of Biologists under the terms of the Creative Commons Attribution License (https://creativecommons.org/licenses/by/4.0/).

How was the vessel wall defined? Was there neointimal remodeling? Was the cross-sectional luminal area decreased, as would be expected from the data in Figs. 4H,I? If yes, either the consequences for the pulmonary circulation were very weak, or there would be some measure of pulmonary hypertension. To that point, (ii) why did the authors examine the cardiac septum? To explore right heart hypertrophy in response to pulmonary hypertension, the Fulton Index would have been employed (the ratio between the weight of the right ventricle versus the left ventricle plus septum). I don't think that the authors can draw the conclusions that they have from the data presented in Figs. 4K,L. (iii) This Reviewer also finds the conclusions drawn from the data presented in Fig. 4J similarly unsupported . One cannot see capillaries in Fig. 4J. What one sees in Fig. 4J is regions of CD3q staining. That is all that can be concluded from Fig. 4J. Overall, while this Reviewer does agree that the consideration of the pulmonary vasculature is indicated to understand the effects reports in the preceding datasets, the conclusions drawn from the datasets presented in Fig. 4 are not supported by the data.

MINOR COMMENTS:

1. The plural form of septum is septa, not septae.

2. This Reviewer is not sure that they would call TGF/beta a cytokine. While TGF-beta certainly does have an impact on cells of the immune system, perhaps it may fall more into the "polypeptide growth factor" grouping?

3. In the quantified data of Fig. 3A (elastin) presented in Fig. 3F, are these really elastin fibres that are being quantified? I believe these are conventionally referred to as elastin foci? This concern occurs again in Fig. 6.

4. The font size in most of the axes labelled in histograms that accompany photomicrographs is too small, allowing for figure size reduction in the final published figures.

5. Might some elements of the figure legends be clarified. I think it is a "Student's t-test" or "Student's t test", not a "Students T test". Sometimes abbreviations are not correctly handled in the figure legends. For example, in the legend to Fig. 7, CMS in the artwork is not defined; and in the legend, MEFs and HLFs are both used without definition.

First revision

Author response to reviewers' comments

Response to reviewers

General editorial points

1. As requested, "track changes" have not been used to indicate where alterations have been made to the manuscript. We have instead highlighted the altered text in a "marked" copy, which has been uploaded in addition to the "clean" copy of our revised manuscript.
2. We have reformatted the manuscript to meet the requirements for publication in *Development*. In particular, the Methods section has been edited to include reagent suppliers, and the "key resources" table has now been moved to the supplements.
3. We have reviewed the figure layout according to the *Development* guidelines to aid readability

Reviewer 1

Point 1: "…their discussion remains very confusing. In parts, there is some concession to the fact that Pdgfrb is not a specific marker of pericytes. Yet in other parts of the revised manuscript the authors persist in claiming that pericytes are progenitors of myofibroblasts. For example, in lines 449-455 they write "Furthermore, while we propose that abnormalities in pericyte differentiation and migration underlie the defective alveologenesis and emphysema in mesenchymal G aq/11 knockout mice, Pdgfrb is expressed by other cell types, including myofibroblasts, fibroblasts and vascular smooth muscle cells. While it is possible that disturbing TGFb signalling in these cell types

© 2023. Published by The Company of Biologists under the terms of the Creative Commons Attribution License (https://creativecommons.org/licenses/by/4.0/).

contributed to the lung phenotype in mesenchymal G aq/11 knockout mice, pericytes are major progenitors for all these cell types, and are therefore likely to have played a primary role in the abnormalities observed". Their claim that pericytes are "major progenitors for myofibroblasts and fibroblasts" is just not substantiated in this paper or elsewhere."

Response 1: We accept that our data cannot definitively confirm which cell types were responsible for the abnormal alveolar development observed in mice lacking $G_{\alpha q/11}$ in *Pdgfrb*-expressing cells. However, previous work has suggested that *Pdgfrb*-expressing cells are located in regions of the lung where pericytes are expected to be found (perivascular areas), and that these cells co-express desmin, another pericyte marker (Henderson et al, Nat Med 2013;19(12):1617-24). Furthermore, *Pdgfrb-Cre/ERT2* mice have been used to study the role of YAP/TAZ signalling in lung morphogenesis, with pericytes thought to be key cells involved in the phenotypes observed (Kato et al, Nat Comms 2018;9:2448). Therefore, the *Pdgfrb-Cre* model should affect pericyte gene expression, and we believe that pericytes may be an important cell type in the phenotype that we observed in mice lacking $G_{\alpha q/11}$ in *Pdgfrb*-expressing cells.

We have modified our discussion to clarify that we can only draw conclusions about the role of *Pdgfrb*-expressing cells, not specifically pericytes, and added more references and discussion to describe which other cell types may be involved (line 478-496).

> "*Pdgfrb-Cre*$^{+/-}$ mice have been used to investigate the role of pericytes in various organ development and disease models (Henderson et al. 2013; Foo et al. 2006; Zaitoun et al. 2019; Gong et al. 2018; S. Wang et al. 2017; He et al. 2020; Ivanova et al. 2021; Zhang et al. 2021; Diéguez-Hurtado et al. 2019; Eilken et al. 2017), including a study that used a GFP reporter mouse to demonstrate *Pdgfrb-Cre*-induced gene recombination in lung pericytes during timepoints relevant to alveolarisation ((Kato et al. 2018). However previous studies have shown that *Pdgfrb-Cre*-induced gene recombination can occur in other cell types, including myofibroblasts induced by injury, fibroblasts, smooth muscle cells, and renal interstitial cells (Henderson et al. 2013; H. Wang et al. 2019; Cuttler et al. 2011; Ulvmar et al. 2016; Gong et al. 2018; Zou et al. 2018; Schiessl et al. 2018). Furthermore, *Pdgfrb* expression may vary at different stages of organ development and cellular differentiation (Salter et al. 2019), and it is possible that altered gene expression in a *Pdgfrb*-expressing precursor cell could influence the characteristics of non-*Pdgfrb*-expressing cells that derive from them. Therefore, while we hypothesise that abnormalities in pericyte activity, differentiation and migration underlie the defective alveologenesis and emphysema in mesenchymal $G_{\alpha q/11}$ knockout mice, the role of other mesenchymal cells in this process cannot be ruled out. However, we can conclude that $G_{\alpha q/11}$ signalling in *Pdgfrb*-expressing cells is important in lung development and homeostasis"

In addition, throughout the revised manuscript, in areas where pericytes are mentioned we have clarified that our work focusses on *Pdgfrb*-expressing cells, not pericytes specifically. However, we do still include the previous literature on the role of pericytes in alveolar development, as these are *Pdgfrb*-expressing cells that are thought to play important roles for lung development, and it is likely that they have been involved in the phenotype observed in our mouse model.

Point 2: "…Moreover, many of the studies are done with MEFs (mouse embryonic fibroblasts) and not with specific populations of mouse (or human) lung fibroblasts."

Response 2: We acknowledge that murine embryonic fibroblasts (MEFs) and primary human lung fibroblasts were used for our in vitro studies rather than specific fibroblast populations. However, our desire was to demonstrate broad generalisability of the stretch-induced TGFb signalling mechanisms. By using both mouse and human cells we have demonstrated the conservation of stretch-induced TGFβ signalling, and the potential roles of protease activity and TGFβ2 specifically, across species enhancing the generalisability of our findings. However, we accept that to suggest that this mechanism is specific to any mesenchymal cell subtype, including pericytes, would require cell-specific experiments and we have added this to the limitations section of our discussion (line 432-434).

> "While the use of MEFs and HLFs does not precisely recapitulate the fibroblast cell populations present during lung development, these data demonstrate conservation of stretch-induced TGFβ signalling in fibroblasts across species."

© 2023. Published by The Company of Biologists under the terms of the Creative Commons Attribution License (https://creativecommons.org/licenses/by/4.0/).

Point 3: "It is just not adequate to talk about "lung fibroblasts" anymore. Without more specific analysis of fibroblast subpopulations and use of additional pericyte driver(s) this revised paper does not advance the field of lung development."

Response 3: We recognise that with the advent of single cell transcriptomics and lineage tracing techniques the understanding of fibroblast subtypes has evolved rapidly. Although it would be interesting to define the specific subset(s) of mesenchymal cells primarily responsible for the mouse phenotypes, and to use these to confirm our in vitro observations, this would be beyond the scope of our study that aimed to understand the role of $G_{\alpha q/11}$ signalling in mesenchymal cells in lung development and homeostasis. Similarly, using an alternative pericyte Cre would add limited extra information. Use of NG2-Cre would be at best "complementary" to Pdgfrb-Cre in the study of pericytes, and as NG2 is expressed by oligodendrocyte precursors and neurones, and previous work has shown that deletion of $G_{\alpha q/11}$ in glial cell precursors (including oligodendrocytes) leads to perinatal death (Wettschureck 2005, Mol Cell Biol 2005 Mar;25(5):1942-8. doi: 10.1128/MCB.25.5.1942-1948.2005.) we do not believe the financial, time or ethical cost to undertake further murine experiments and sequencing studies would be justifiable for the extra information obtained. Therefore, we have edited the discussion to highlight that the lack of fibroblast subtype definition and lineage tracing could be a limitation of this work (line 476-478).

> "The absence of detailed lineage tracing or single cell RNA sequencing of the Pdgfrb-Cre$^{+/-}$;Gnaq$^{fl/fl}$;Gna11$^{-/-}$ mouse lungs means it cannot be confirmed that the abnormalities were driven by any particular mesenchymal cell subtype"

We have reviewed our manuscript to ensure that the fact that *Pdgfrb* is not a pericyte-specific marker is emphasised, and refer to *Pdgfrb*-expressing cells rather than pericytes when referring to our own data.

Point 4: "The authors state that they use Pdgfrb-Cre/ERT2+/- mice (Laboratories). Do they mean Jackson Laboratories? They need to cite the original generation of these mice."

We thank the reviewer for pointing out this typographical error. The *Pdgfrb-Cre/ERT2+/-* mice were purchased from Jackson Laboratories, and we have corrected this in the manuscript. We have also referenced the original generation of these animals.

<u>Reviewer 2</u>

Point 1: "I still think they are missing a trick by underplaying the significance of defective elastin deposition and distribution in their model. But I suppose that's up to them. Certainly elastin is stretchy and has a lot to do with force distribution and recoil in springy tissues like the lung."

We agree with the reviewer that defective elastin production is likely to be a key contributor to the lung phenotype that we observed in the lungs of mice lacking mesenchymal $G_{\alpha q/11}$. We have added to the section that discusses the finding of altered elastin distribution and deposition in our mouse models to describe the potential roles of this in the detection and generation of mechanical forces that may be relevant to our model, and to emphasise that this is a fascinating area that requires further study (line 374-379).

> "In addition, as elastin is a key factor governing lung compliance (Hilgendorff et al. 2012; Dolhnikoff, Mauad, and Ludwig 1999), and mechanical forces themselves may alter availability of elastin binding sites (Jesudason et al. 2010; Suki et al. 2012), elastin may influence the response to stretch-related forces. Therefore, the impact of mesenchymal $G_{\alpha q/11}$ deletion on elastin deposition and distribution may influence the response to and generation of mechanical forces within the lungs."

<u>Reviewer 3</u>

Major comments

Point 1. Might the authors comment on how restricted genetic recombination directed by the Pdgfrb-Cre driver line? Is the cellular distribution of the recombination activity of the driver

© 2023. Published by The Company of Biologists under the terms of the Creative Commons Attribution License (https://creativecommons.org/licenses/by/4.0/).

line restricted to the cell-types addressed in the report, in the developing lung? Might an mTmG panel be available for this time-point for the relevant mouse strains (perhaps this is already available from other strain characterisation reports, which could be referenced here). Alternatively, can an additional short discussion be added to the manuscript to address this point?

Response 1:
We unfortunately do not have data using a fluorescent reporter mouse to indicate the cellular distribution of recombination activity. However, other groups have published this important work. Of particular relevance to our study, Kato et al (2018) used a GFP reporter mouse to demonstrate *Pdgfrb-Cre*-induced gene recombination in pericytes during timepoints relevant to alveolarisation and our study (at P7, P21, and P50), but not PDGFRα+ fibroblasts and alpha smooth muscle actin (αSMA)+ bronchial smooth muscle cells or myofibroblasts. We have added a section to our discussion to address this point, and the potential limitations of our study that relate to it (line 478-496).

> *"Pdgfrb-Cre$^{+/-}$* mice have previously been used to investigate the role of pericytes in various organ development and disease models (Henderson et al. 2013; Foo et al. 2006; Zaitoun et al. 2019; Gong et al. 2018; S. Wang et al. 2017; He et al. 2020; Ivanova et al. 2021; Zhang et al. 2021; Diéguez-Hurtado et al. 2019; Eilken et al. 2017), including a study that used a GFP reporter mouse to demonstrate *Pdgfrb-Cre*-induced gene recombination in lung pericytes during timepoints relevant to alveolarisation ((Kato et al. 2018). However previous studies have shown that *Pdgfrb-Cre*-induced gene recombination can occur in other cell types, including myofibroblasts induced by injury, fibroblasts, smooth muscle cells, and renal interstitial cells (Henderson et al. 2013; H. Wang et al. 2019; Cuttler et al. 2011; Ulvmar et al. 2016; Gong et al. 2018; Zou et al. 2018; Schiessl et al. 2018). Furthermore, *Pdgfrb* expression may vary at different stages of organ development and cellular differentiation (Salter et al. 2019), and it is possible that altered gene expression in a *Pdgfrb*-expressing precursor cell could influence the differentiation and characteristics of non-*Pdgfrb*-expressing cells that derive from them. Therefore, while we hypothesise that abnormalities in pericyte activity, differentiation and migration underlie the defective alveologenesis and emphysema in mesenchymal $G_{\alpha q/11}$ knockout mice, the role of other mesenchymal cells in this process cannot be ruled out. However, we can conclude that $G_{\alpha q/11}$ signalling in *Pdgfrb*-expressing cells is important in lung development and homeostasis."

Point 2. Throughout, the authors have selected Pdgfrb-Cre-/-,Gnaqfl/fl,Gna11-/- mice for their control groups. This is understandable, given that the floxed Gnaq allele cannot be recombined in the absence of Cre. However, this control group does not permit consideration of two potentially important confounding variables in the control groups: the presence of a Gnaq floxed allele, and the lack of Cre. Whether the introduction of the floxed Gnaq allele has any impact on Gnaq expression in the absence of Cre, compared with mice carrying an unfloxed Gnaq allele is not known. Also, the impact of overexpression of Cre in the Pdgfrb-Cre-/+ "controls" is also not known, Do the authors have any information about how lung structure looks, or Gnaq is expressed, comparing their Pdgfrb-Cre-/-,Gnaqfl/fl,Gna11-/- control strain with a Pdgfrb-Cre-/+,Gnaq+/+,Gna11-/- strain? In not, might the strain selection be rationalized in the text?

Response 2: We thank the reviewer for these very important points, namely the effects of expression of the floxed Gnaq allele in the absence of a Cre driver and the potential for Cre 'toxicity' confounding our results. We have analysed histology from mice of all eight possible genotypes that arose from the constitutive mesenchymal $G_{\alpha q/11}$ knockout mouse model, which includes mice homozygous for the floxed *Gnaq* allele in the absence of Cre expression (*Pdgfrb-Cre$^{-/-}$;Gnaq$^{fl/fl}$;Gna11$^{-/-}$* and *Pdgfrb-Cre$^{-/-}$;Gnaq$^{fl/fl}$;Gna11$^{+/-}$*) and mice that express Cre but still have one functional *Gnaq* allele (*Pdgfrb-Cre$^{+/-}$;Gnaq$^{+/fl}$;Gna11$^{-/-}$* and *Pdgfrb-Cre$^{+/-}$;Gnaq$^{+/fl}$;Gna11$^{+/-}$*). Histological appearances of mice expressing the floxed *Gnaq* allele in the absence of the Cre driver were normal. We confirmed this by analysing the mean linear intercept distance, measuring alveolar wall thickness, and quantifying secondary crests of all eight possible genotypes from this breeding strategy. Indeed all seven genotypes 'control' genotypes were normal. We have added a

© 2023. Published by The Company of Biologists under the terms of the Creative Commons Attribution License (https://creativecommons.org/licenses/by/4.0/).

supplementary figure (supp figure 1), and a section to the results to demonstrate this (line 143-150).

> "Mice with at least one expressed *Gnaq* or *Gna11* allele (i.e. any genotype other than *Pdgfrb-Cre*$^{+/-}$*;Gnaq*$^{fl/fl}$*;Gna11*$^{-/-}$) had normal lung histological appearances and similar morphometric measurements that differed from those of *Pdgfrb-Cre*$^{+/-}$*;Gnaq*$^{fl/fl}$*;Gna11*$^{-/-}$ mice (**Figure S1**), indicating that a complete absence of *Gnaq* and *Gna11* in mesenchymal cells is required for the abnormal lung phenotype to be observed. These data also demonstrate that Cre expression or presence of the floxed Gnaq alleles alone do not influence the lung phenotype observed in *Pdgfrb-Cre*$^{+/-}$*;Gnaq*$^{fl/fl}$*;Gna11*$^{-/-}$ mice."

The mesenchymal $G_{\alpha q/11}$ knockout mouse, *Pdgfrb-Cre*$^{+/-}$*;Gnaq*$^{fl/fl}$*;Gna11*$^{-/-}$ had substantial differences in the lung histological appearances compared with all the other possible genotypes, which express at least one functional *Gnaq* or *Gna11* allele. This, in combination with the body weight data shown in Figure 1, suggests that there must be a complete absence of functional *Gnaq* or *Gna11* alleles in mesenchymal cells for the phenotype to be observed, and suggest there is no major functional consequence of 'floxed' Gnaq (consistent with previous findings, John et al Sci Signal 2016;9(451):ra104).

We acknowledge that Cre 'toxicity' can occur, however we are reassured that we did not see any effect of Cre overexpression in our studies as there was no evidence of organ abnormality or weight change in mice that were expressing Cre in the absence of Gnaq$^{fl/fl}$. These data are now included in supplementary figure 1.

We used *Pgdfrb-Cre*$^{-/-}$*:Gnaq*$^{fl/fl}$*;Gna11*$^{-/-}$ mice as our control group, as it is known that *Gna11*$^{-/-}$ mice have no lung phenotype (John et al Sci Signal 2016;9(451):ra104; Offermanns 1998 EMBO J 1998;17(5):4303-4312), and our data show no effect of the Gnaq floxed allele. This also allowed us to use littermate controls which were the same age and held under the same conditions as mice with the genotype of interest (*Pdgfrb-Cre*$^{+/-}$*:Gnaq*$^{fl/fl}$*;Gna11*$^{-/-}$). We have clarified which mice were used as controls in the methods (line 611-613 and 620-622) and results (line 129-134) sections, and have added a rationale for the control group used.

> "*Gnaq*$^{fl/fl}$*;Gna11*$^{-/-}$ mice develop normally and do not express a phenotype (John et al. 2016), therefore *Pdgfrb-Cre*$^{-/-}$*;Gnaq*$^{fl/fl}$*;Gna11*$^{-/-}$ littermates were used as controls for all analyses to ensure that control mice had an identical genotype to the mesenchymal $G_{\alpha q/11}$ knockout mice other than Cre expression, and to allow the use of age-matched littermate controls. From here, mice with the *Pdgfrb-Cre*$^{-/-}$*;Gnaq*$^{fl/fl}$*;Gna11*$^{-/-}$ genotype will be referred to as *Gna11*$^{-/-}$ controls.

3. In the Pdgfrb-Cre+/-,Gnaqfl/fl,Gna11-/- mice, lungs were considered heavier at P14, compared to Gna11+/+ mice. Two questions: was the control comparison made with Gna11+/+ mice, or with Pdgfrb-Cre+/- mice, or with Gna+/+ as stated (p. 5). (i) In the case of the latter, might the presence of Pdgfrb-Cre+/- with an unexpected off-target effect have contributed to this phenomenon in the experimental group, and (ii) irrespective of the Pdgfrb-Cre status of the control group, could this increased weight be due to fluid accumulation in the lungs (i.e. was alveolar or interstitial oedema present?).

Response 3: As stated above The comparison of relative lung weights in Figure 2 is between *Pdgfrb-Cre*$^{-/-}$*:Gnaq*$^{fl/fl}$*;Gna11*$^{-/-}$ controls (also referred to as *Gna11*$^{-/-}$ for ease of readability) and *Pdgfrb-Cre*$^{+/-}$*:Gnaq*$^{fl/fl}$*;Gna11*$^{-/-}$ (mesenchymal $G_{\alpha q/11}$ knockout) mice, with the latter having a higher relative lung weight. We acknowledge that Cre 'toxicity' can occur however we are reassured that we did not see any effect of Cre overexpression in our studies as there was no evidence of organ abnormality or weight change in mice that were expressing Cre in the absence of Gnaq$^{fl/fl}$. We have presented relative lung weight data from all eight possible genotypes from our breeding strategy (supplementary figure 1), and demonstrated that the mesenchymal *Pdgfrb-Cre*$^{-/-}$*:Gnaq*$^{fl/fl}$*;Gna11*$^{-/-}$ mice had the highest relative lung weights compared with the other genotypes, and that there is no effect of Cre expression alone on this parameter. We have added a section to the results to explain this (line 161-162).

> "This trend of increased lung weight in *Pdgfrb-Cre*$^{+/-}$*;Gnaq*$^{fl/fl}$*;Gna11*$^{-/-}$ mice was also observed when compared to all possible genotypes possible from this breeding strategy (**Figure S1**)."

© 2023. Published by The Company of Biologists under the terms of the Creative Commons Attribution License (https://creativecommons.org/licenses/by/4.0/).

In response to point ii, we agree with the reviewer that interstitial oedema could increase the relative lung weight and have added this as a potential explanation to our results (line 160) and discussion (line 401-403) sections.

> Results: “Finally, *Pdgfrb-Cre*$^{+/-}$*;Gnaq*$^{fl/fl}$*;Gna11*$^{-/-}$ lungs were heavier relative to total body weight compared with lungs from *Gna11*$^{-/-}$ mice (16.5 vs 14.3mg/g total body weight, p<0.01, **Figure 2G**), which could be due to elevated lung density or interstitial oedema in these animals”
> Discussion: “Furthermore, the observed increased lung weights in *Pdgfrb-Cre*$^{+/-}$*;Gnaq*$^{fl/fl}$*;Gna11*$^{-/-}$ mice could be linked to interstitial oedema and cardiac malfunction”

Point 4. Given the emphasis on pericytes in the text, the consideration of the lung vascular structure is well-taken (primarily presented in Fig. 4). Along these lines: the consideration of lung vascular structure rests on interpretation of immunohistochemical data, primarily, the estimation of maximum and minimum wall thickness (Fig. 4 A,J) and visual inspection of the cardiac septum (Fig. 4K), leading the authors to conclude that lung vessels were markedly abnormal (p. 6) and that cardiac septal wall thickness is unchanged, which the authors apparently took to mean that there was no evidence of pulmonary hypertension. Additionally, the capillaries were considered to be normal in “appearance”. I find this data set in the manuscript to very thinly support the conclusions drawn from the data.

(i) There are well-established technique to study lung (indeed, any) vascular structure, and in terms of lung pathology, standard morphometric techniques to study vascular wall thickness have not been employed. Can the author provide other evidence of change sin lung conducting vessel structure. The immunohistochemically images provide little clarity on this point. Data in Figs.4H,I suggest increased minimum and maximum vessel wall thickness. Are we talking about the vascular media? How was the vessel wall defined? Was there neointimal remodeling? Was the cross-sectional luminal area decreased, as would be expected from the data in Figs. 4H,I? If yes, either the consequences for the pulmonary circulation were very weak, or there would be some measure of pulmonary hypertension.

Response 4 (i):

Many thanks to the reviewer for these helpful comments. In response to these comments we have re-analysed our data in figure 4 using a standard method of calculating vessel wall thickness which corrects for the vessel size (Hoshikawa et al ALRCCM 2001;164:314-318). Vessel wall thickness was defined using the formula for vessel wall thickness = (external vessel diameter- internal vessel diameter)/ external diameter). When using this method, our data suggest increased peripheral pulmonary vessel wall thickness in mesenchymal $G_{\alpha q/11}$ knockout mice. This method measures the entire vessel wall, but the images suggest that this is driven by thickening of the vascular media. We have updated our methods to fully describe this approach (line 729-740).

> “The external vessel diameter (ED) was defined as the distance from the outermost aspect of the external wall of a vessel to the outermost aspect of the opposite wall, traversing the centre of the vessel lumen. The internal vessel diameter (ID) was defined as the distance from the innermost aspect of the vessel wall to the innermost aspect of the opposite vessel wall, traversing the centre of the vessel lumen. The following equations were used to calculate the vessel wall thickness (VWT) and internal vessel lumen diameter relative to total vessel diameter;
>
> VWT = (ED-ID) / ED
>
> Internal vessel diameter = ID / ED”

We have measured cross-sectional luminal area by measuring the internal luminal diameter relative to whole vessel diameter, and this was decreased in the mesenchymal $G_{\alpha q/11}$ knockout mice. When corrected for total vessel diameter, our data suggest that the luminal diameter of the pulmonary vessels in mesenchymal $G_{\alpha q/11}$ knockout mice is reduced compared with control animals.
Although the data do not show complete vessel occlusion, this could be consistent with pulmonary hypertension. The αSMA staining shown in Figure 4 shows that there may be muscularisation of the

© 2023. Published by The Company of Biologists under the terms of the Creative Commons Attribution License (https://creativecommons.org/licenses/by/4.0/).

vessel walls in the abnormal peripheral pulmonary vessels, which supports this hypothesis. However, we do not see any evidence of intimal or adventitial fibrosis or neointimal remodelling which can be seen in pulmonary hypertension, as there was not an increase in collagen or elastin staining in the mesenchymal $G_{\alpha q/11}$ knockout mouse lungs.

We accept that we do not have physiological data to support the presence of pulmonary hypertension and can therefore only present hypotheses for the interesting morphological changes observed. We have edited our discussion of the pulmonary vasculature to reflect this, and have reduced the emphasis on pulmonary hypertension, as there are a multitude of potential causes for the abnormalities observed that we cannot conclude further from our data.

Point 4 (ii) why did the authors examine the cardiac septum? To explore right heart hypertrophy in response to pulmonary hypertension, the Fulton Index would have been employed (the ratio between the weight of the right ventricle versus the left ventricle plus septum). I don't think that the authors can draw the conclusions that they have from the data presented in Figs. 4K,L

Response 4 (ii):

As right heart failure is an important consequence of pulmonary abnormalities such as pulmonary hypertension, we felt that some evaluation of the heart was important. Unfortunately, all the cardiac samples from this study are now paraffin embedded and partially cut, so it is not possible to measure the Fulton index for this mouse study. While our method of measuring the right ventricle: left ventricle wall thickness ratio is not ideal, it was the best approach that we could take given the samples available. We have added this limitation to our discussion (line 387-403), and have reduced the emphasis on pulmonary hypertension when discussing this data.

> "*Pdgfrb-Cre*$^{+/-}$*;Gnaq*$^{fl/fl}$*;Gna11*$^{-/-}$ mouse lungs contained abnormal peripheral pulmonary vessels, with thickened vessel walls and reduced lumen diameter associated with muscularisation of the media (indicated by αSMA staining). These findings could be explained by pulmonary arterial hypertension (PAH), which could relate to hypoxaemia secondary to the profound pulmonary defects, in combination with disturbed GPCR signalling, resulting in vascular remodelling (Patel et al. 2018; Cheng et al. 2012). However, cardiac histology did not show thickening of the right ventricular wall in *Pdgfrb-Cre*$^{+/-}$*;Gnaq*$^{fl/fl}$*;Gna11*$^{-/-}$ mice, neither was any intimal or adventitial fibrosis observed, findings inconsistent with substantial PAH. A limitation of this study is that physiological such as the Fulton index to assess for right ventricular hypertrophy were not possible and so it is not possible to determine conclusively whether there was any PAH. An alternative explanation for the abnormal vasculature in *Pdgfrb-Cre*$^{+/-}$*;Gnaq*$^{fl/fl}$*;Gna11*$^{-/-}$ mice could be that altered activity of *Pdgfrb*-expressing cells influences vascular development or the growth, differentiation, and activity of constituent cells such as vascular smooth muscle cells. Furthermore, the observed increased lung weights in *Pdgfrb-Cre*$^{+/-}$*;Gnaq*$^{fl/fl}$*;Gna11*$^{-/-}$ mice could be linked to interstitial oedema and cardiac malfunction. However, it is not currently possible to define the precise cause of the vascular abnormalities observed.

Point 4 (iii) This Reviewer also finds the conclusions drawn from the data presented in Fig. 4J similarly unsupported . One cannot see capillaries in Fig. 4J. What one sees in Fig. 4J is regions of CD3q staining. That is all that can be concluded from Fig. 4J. Overall, while this Reviewer does agree that the consideration of the pulmonary vasculature is indicated to understand the effects reports in the preceding datasets, the conclusions drawn from the datasets presented in Fig. 4 are not supported by the data.

Response 4(iii): We are grateful that the reviewer acknowledges the importance of considering the pulmonary vasculature in our studies. We also accept that that we only show that CD31 staining around alveoli and have edited the manuscript to reflect this (line 207-210).

> "CD31 staining of the alveoli of *Pdgfrb-Cre*$^{+/-}$*;Gnaq*$^{fl/fl}$*;Gna11*$^{-/-}$ lungs had a similar appearance to those seen in *Gna11*$^{-/-}$ lungs at high magnification (**Figure 4J**). This argues against there being gross abnormality of the small alveolar vessels, however this cannot be completely ruled out."

© 2023. Published by The Company of Biologists under the terms of the Creative Commons Attribution License (https://creativecommons.org/licenses/by/4.0/).

Minor comments

Point 1. The plural form of septum is septa, not septae.

Response 1: This has been corrected throughout the manuscript.

Point 2. This Reviewer is not sure that they would call TGF/beta a cytokine. While TGF-beta certainly does have an impact on cells of the immune system, perhaps it may fall more into the "polypeptide growth factor" grouping?

Response 2: While TGFβ is often referred to as a cytokine and can influence immune cell function, we agree that this description may not be the best fit for the biological effects of TGFβ that we are describing. We have therefore changed "cytokine" to "growth factor".

Point 3. In the quantified data of Fig. 3A (elastin) presented in Fig. 3F, are these really elastin fibres that are being quantified? I believe these are conventionally referred to as elastin foci? This concern occurs again in Fig. 6.

Response 3: We thank the reviewer for pointing this out. We have edited the manuscript and figure legends to correct this.

Point 4. The font size in most of the axes labelled in histograms that accompany photomicrographs is too small, allowing for figure size reduction in the final published figures.

Response 4: All of the figures have been reviewed and altered where necessary to ensure that the text in the figures is clear and to ensure that the labelling of histograms is consistent. The typographical errors in the figure labels and legends have been corrected.

Point 5. Might some elements of the figure legends be clarified. I think it is a "Student's t-test" or "Student's t test", not a "Students T test". Sometimes abbreviations are not correctly handled in the figure legends. For example, in the legend to Fig. 7, CMS in the artwork is not defined; and in the legend, MEFs and HLFs are both used without definition.

Response 5: Many thanks for this helpful feedback. We have edited the figures and figure legends to ensure that abbreviations are fully defined, and to correct grammatical errors.

<u>Second decision letter</u>

MS ID#: DEVELOP/2022/201046

MS TITLE: Stretch Regulates Alveologenesis and Homeostasis Via Mesenchymal $G_{\alpha q/11}$-mediated TGFβ2 Activation

AUTHORS: Amanda T Goodwin, Alison E John, Chitra Joseph, Anthony Habgood, Amanda L Tatler, Katalin Susztak, Matthew Palmer, Stefan Offermanns, Neil C Henderson, and R Gisli Jenkins
ARTICLE TYPE: Research Article

I am happy to tell you that your manuscript has been accepted for publication in Development, pending our standard ethics checks.

<u>Reviewer 2</u>

Advance summary and potential significance to field

© 2023. Published by The Company of Biologists under the terms of the Creative Commons Attribution License (https://creativecommons.org/licenses/by/4.0/).

This paper introduces the important concept that intrapulmonary stretch plays a key role in the process of alveoarization, suggesting that existing theories of alveolarization such as "erection" of alveolar septae need to be carefully reconsidered.

Comments for the author

The authors have made a major effort to respond to all 3 reviewers.
Reviewer 2 is satisfied with their revisions

Reviewer 3

Advance summary and potential significance to field

Teh core content fo the manuscript has not changed, as such, my original assessment of the advance made in this paper and its potential significance to the field remains unchanged.

Comments for the author

I have no further Comments for the authors.

© 2023. Published by The Company of Biologists under the terms of the Creative Commons Attribution License (https://creativecommons.org/licenses/by/4.0/).
